# Novel oncolytic vaccinia virus armed with interleukin-27 is a potential therapeutic agent for the treatment of murine pancreatic cancer

**DOI:** 10.1136/jitc-2024-010341

**Published:** 2025-05-11

**Authors:** Yangyang Jia, Yanru Wang, Guanghao Zhao, Yong Yang, Wenyi Yan, Ruimin Wang, Bing Han, Lihong Wang, Zhe Zhang, Lijuan Chen, Nicholas R Lemoine, Louisa S Chard Dunmall, Pengju Wang, Yaohe Wang

**Affiliations:** 1 Sino-British Research Centre for Molecular Oncology, National Centre for International Research in Cell and Gene Therapy, State Key Laboratory of Metabolic Dysregulation & the Prevention and Treatment of Esophageal Cancer, School of Basic Medical Sciences, Academy of Medical Sciences, Zhengzhou University, Zhengzhou, China; 2 Department of Oncology, Air Force Medical Center, PLA, Beijing, China; 3 Department of Gastroenterology, The Second Affiliated Hospital of Zhengzhou University, Zhengzhou, China; 4 Department of Oncology, Henan International Joint Laboratory of Lung Cancer Biology and Therapeutics, the Affiliated Cancer Hospital of Zhengzhou University & Henan Cancer Hospital, Zhengzhou, China; 5 Centre for Cancer Biomarkers & Biotherapeutics, Barts Cancer Institute, Queen Mary University of London, London, EC1M 6BQ, UK

**Keywords:** Oncolytic virus, Cytokine, Immunotherapy, Gastrointestinal Cancer

## Abstract

**Background:**

Pancreatic cancer has a complex immunosuppressive tumor microenvironment (TME), which is highly resistant to conventional therapies and emerging cancer immunotherapies. Oncolytic viruses are multifaceted killers of malignant tumors, which can selectively infect, replicate in and lyse tumor cells, release tumor-associated antigens to stimulate specific antitumor immune responses, and recruit immune cells into the TME, turning “cold” tumors “hot”. Here, we report a novel *vaccinia virus* (VV), VVLΔTKΔN1LΔA41L (with deletion of thymidine kinase (TK), N1L, and A41L genes) armed with interleukin 27 (IL-27), that can cure established tumors and promote long-term antitumor immunity in murine pancreatic cancer tumor models.

**Methods:**

A novel oncolytic VV with deletion of the TK, N1L, and A41L genes, and expression of the red fluorescent protein (RFP) gene (VVL-TD-RFP) was constructed using CRISPR-Cas9-based homologous recombination. This virus was armed with IL-27, creating VVL-TD-IL-27. The characteristics of these viruses were evaluated *in vitro* using viral replication assays, cytotoxicity assays and ELISA. The antitumor effects of VVL-TD-IL-27 were evaluated using a variety of pancreatic cancer tumor models *in vivo*, and the mechanisms of antitumor effects were explored using flow cytometry, immunohistochemistry, ELISA and quantitative PCR.

**Results:**

VVL-TD-RFP cured 71.4% of tumor-bearing mice, compared with 14.3% of animals treated with VVLΔTKΔN1L that does not have an A41L gene deletion. Efficacy was mainly dependent on elevated dendritic cell (DC) populations, activation of DC, CD86^+^ DC, and CD8^+^ effector memory T cells in the TME. Efficacy was further enhanced by arming VVL-TD-RFP with IL-27, which resulted in a cure rate of 100% and promoted long-term antitumor immunity. VVL-TD-IL-27 treatment increased the proportion of CD8^+^ TEM and decreased the proportion of regulatory T cells and macrophages in tumor tissues. It also polarized macrophages to an M1 phenotype *in vivo*. Furthermore, IL-27 exhibits strong anti-angiogenic effects.

**Conclusions:**

VVL-TD-mIL-27 is a potential immunotherapy agent for the treatment of pancreatic cancer, and a clinical study of this virus is warranted.

**WHAT IS ALREADY KNOWN ON THIS TOPIC**

- It has been demonstrated that the oncolytic *vaccinia virus* Lister strain (VVL) can effectively replicate and express therapeutic genes in a hypoxic environment, making it an excellent vector for the treatment of hypoxic tumors such as pancreatic cancer. A VVL (called VVLΔTKΔN1L) with thymidine kinase (TK) and N1L gene deletions has exhibited strong safety and antitumor efficacy in different tumor models but cannot completely eradicate the established tumors.

**WHAT THIS STUDY ADDS**

- To further optimize the viral vector, we deleted the A41L gene of the VVL and constructed a novel VVL backbone VVL-TD-RFP. *In vivo*, VVL-TD-RFP increased tumor clearance compared with VVLΔTKΔN1L. Mechanistic studies revealed that the A41L deletion could significantly increase dendritic cell subsets and CD8^+^ effector memory T cells in tumor tissues. To further increase the antitumor effect, we armed the VVL-TD-RFP with interleukin (IL)-27 to create VVL-TD-IL-27. VVL-TD-IL-27 could effectively replicate and kill tumor cells, and expresses IL-27 *in vitro*. VVL-TD-IL-27 further enhanced the antitumor effect compared with VVL-TD-RFP and induced long-term antitumor immune memory by reducing the proportion of regulatory T cells and macrophages in tumor tissues, promoting the polarization of M2 to M1 macrophages, and inhibiting tumor angiogenesis.

**HOW THIS STUDY MIGHT AFFECT RESEARCH, PRACTICE OR POLICY**

- This study suggests that VVL-TD-RFP is a promising new backbone for the development of cancer therapeutics. Arming this virus with IL-27 (VVL-TD-IL-27) created a safe, yet potent biological agent for cancer treatment, which could provide new treatment options for pancreatic cancer and other patients with tumor.

## Introduction

 Pancreatic cancer (PaCa) is one of the deadliest malignancies. It is the seventh leading cause of cancer death worldwide.^1^ Currently, the primary therapeutic strategies for PaCa are surgery and chemotherapy. However, only 15–20% of patients can undergo surgery at diagnosis.^2^ The standard chemotherapy regimen for PaCa is FOLFIRINOX (fluorouracil, leucovorin, irinotecan, oxaliplatin), but many patients are resistant to chemotherapy, resulting in a 5-year overall survival rate of only 13%, the lowest of all cancers.^3^ This is because PaCa has a challenging “immune privileged” tumor microenvironment (TME) characterized by numerous fibroblasts and dense extracellular matrix forming physical barriers, which limits drug penetration and leads to poor infiltration of immune cells.^4^ More importantly, the hypoxic characteristic of PaCa promotes its invasiveness and treatment-resistant phenotype.^5^ A new therapeutic strategy is therefore urgently needed to overcome the “cold” TME that is not cross-resistant with conventional therapies.

Oncolytic virus therapy (OVT) is a novel and promising cancer immunotherapy, which uses natural or genetically engineered oncolytic viruses (OVs) to selectively replicate in tumor cells without damaging normal cells. OVs mediate their antitumor effect through multipronged mechanisms. First, selective infection, replication, and lysis of cancer cells *in situ*. Second, the release of tumor-associated antigens, which is regarded as an *in situ* vaccine to induce a systemic and specific antitumor immune response. Third, the release of cytokines, danger-associated molecular pattern signals, and viral pathogen-associated molecular patterns to recruit immune cells into the TME,^6^ leading to the remodeling of an immunologically “cold” TME into a “hot” one. Importantly, OVs can be genetically modified to express therapeutic genes that enhance antitumor immune responses. Therefore, OVs provide a new platform for cancer patients whose tumors are resistant to conventional therapies.

*Vaccinia virus* (VV) is a double-stranded linear DNA virus approximately 192 kb in length that can encode about 200 genes.^7^ Its safety has been determined through its extensive use in the smallpox eradication campaign. VV has many advantages compared with other OVs. The viral life cycle occurs in the cytoplasm, mitigating a risk of genome integration into host chromosomes.^7^ VV has a short life cycle that leads to lysis of tumor cells in 12–48 hours.^8^ The virus enters the cell by membrane fusion, which allows receptor-independent infection of a wide range of tumor cells.^9^ Foreign genes up to 25 kb can be inserted,^10^ and unlike most other OVs, VV can be successfully delivered intravenously, allowing simultaneous targeting of primary, disseminated and metastatic tumors.^11^ Importantly, we found that hypoxia, which is often a feature of treatment-resistant tumors, does not affect VV Lister (VVL) strain replication, cytotoxicity, and transgene expression.^12^ Therefore, VVL strain is a potential therapeutic vector for targeting PaCa and other hypoxic tumors.

Recently, we described a novel VVLΔTKΔN1L with thymidine kinase (TK) and N1L gene deletion, which demonstrated a stronger antitumor effect and activated stronger antitumor immunity in different tumor models, compared with N1L-intact VVLΔTK.^13^ Here, we further optimized the virus vector by deleting the A41L gene and constructed VVLΔTKΔN1LΔA41L-RFP (hereafter referred to as VVL-TD-RFP). Chemokines are part of the innate immune response that directs the migration of immune cells to areas of infection and inflammation.^14^ The A41L gene encodes a 30 kDa immunomodulatory protein that shares sequence similarity to viral CC chemokine inhibitor (vCCI), which interferes with chemokine-mediated migration of leukocytes to the site of infection, thereby affecting the host response to VV infection.^15^ Genome sequencing showed that A41L is a highly conserved gene encoded by all studied orthopoxvirus species and strains (more than 70 in total).^15^ Nonetheless, it is a non-essential gene for virus replication. VV Western Reserve (WR) strain lacking the A41L gene formed normal-sized plaques and had the same titer as the wild-type virus. In addition, A41L gene deletion increased immunopathology and the infiltration of inflammatory cells into the infected area in a rabbit intradermal infection model.^16^ It is reported that the immunogenicity and vaccine efficacy of WR and modified *vaccinia virus* Ankara (MVA) strains with an A41L gene deletion are enhanced and this results in an increase in CD8^+^ T cells infiltration induced by the A41L gene deletion.^17^ Here, we demonstrate that intratumoral injection of A41L-deleted VVL-TD-RFP could improve the cure rate of tumor-bearing mice. Compared with A41L-intact VVLΔTKΔN1L, the infiltration of CD4^+^ T and natural killer (NK) cells in TME and the proportion of dendritic cell (DC), activated DC, CD86^+^ DC and CD8^+^ T cells in tumor tissues increased.

Interleukin 12 (IL-12) is a powerful agent for antitumor immunotherapy. However, its excessive toxicity limits its clinical application.^18^ IL-27 is a new member of the IL-12 family, which was discovered and named in 2002 and has potent antitumor activity.^19 20^ IL-27 exerts its antitumor effects through a variety of mechanisms, including enhancing the survival of tumor antigen-specific CD8^+^ T cells,^21^ promoting the activation of NK cells,^22^ inhibiting angiogenesis,^23^ increasing M1-polarized tumor-associated macrophages,^24^ and directly suppressing tumor cells proliferation.^25^ Recently, it has been reported that an adeno-associated virus expressing IL-27 (AAV-IL-27) could induce the depletion of regulatory T (Treg) cells to enhance the efficacy of cancer immunotherapy.^26^ In the present study, we armed the virus with human or murine IL-27 to construct VVLΔTKΔN1LΔA41L-h/mIL-27 (hereafter referred to as VVL-TD-h/mIL-27) and proved that VVL-TD-mIL-27 has a strong antitumor effect, which can achieve up to 100% tumor clearance and the induction of long-term antitumor immune responses.

## Materials and methods

### Viruses

The construction of VVLΔTKΔN1L was described previously.^13^ pUC57-A41L (pA41L) shuttle vector (A41L left arm-H5-RFP-H5-A41L right arm) was synthesized by GENEWIZ (Jiangsu, China) and contains SalI and NheI restriction enzyme sites. pUC57-hIL-27 (containing the human IL-27 cytokine) and pUC57-mIL-27 (containing the murine IL-27 cytokine) were synthesized by GENEWIZ (Jiangsu, China) with SalI and NheI restriction sites at either ends of the gene sequence. IL-27 comprises an EBI3 subunit and a p28 subunit linked by an elastic peptide (VPGVGVPGVG). pUC57-A41L and pUC57-IL-27 (containing the human or murine IL-27 cytokine) were digested with SalI and NheI simultaneously and then ligated with T4 ligase to construct pUC57-A41L-hIL-27 (pA41L-hIL-27) and pUC57-A41L-mIL-27 (pA41L-mIL-27) shuttle vectors (A41L left arm-H5-RFP-H5-IL-27-A41L right arm). CRISPR-Cas9-based homologous recombination^27^ was used to construct the VVLΔTKΔN1LΔA41L-RFP and VVLΔTKΔN1LΔA41L-IL-27 (containing the human or murine IL-27 cytokine) using VVLΔTKΔN1L. PCR and DNA sequencing were used to confirm successful construction, and the virus was expanded and purified from CV1 cells. Virus titer was determined by measuring the median tissue culture infective dose (TCID50) on indicator CV1 cells. Cytopathic effect was determined by light microscopy 8 days after infection. The Reed-Muench mathematical method was used to calculate the TCID50 value.^28^ For mouse studies, VVL-TD-mIL-27 armed with murine IL-27 was used. For hamster studies, VVL-TD-hIL-27 armed with human IL-27 was used.

### In vivo studies

All animal studies were approved by the Animal Welfare and Research Ethics Committee of Zhengzhou University (Zhengzhou, China) for the project 2019YFC1316101PW (ID V3A02022000001) and followed the ARRIVE reporting guidelines.^29^ 2×10^6^ DT6606 or 1×10^6^ TB11381 cells were implanted subcutaneously into one flank of 6–7-week-old immune-competent male or female C57BL/6 mice. When the tumor volume reached 100 mm^3^ mice were stratified into three or four groups and received intratumoral injections of 1×10^8^ PFU (plaque forming unit) of virus (VVLΔTKΔN1L, VVL-TD-RFP, or VVL-TD-mIL-27) or phosphate-buffered saline (PBS) on days 0, 2, 4 and 1×10^8^ PFU of virus on days 11, 13 and 15 (two biological repeats were carried out) or 1×10^8^ PFU of virus on days 0, 2, and 4 (one biological repeat was carried out).

For the intraperitoneal dissemination model, 1×10^7^ SHPC6 cells were inoculated into the lower right peritoneal cavity of 6–7-week-old immune-competent female Syrian hamsters. 4 days later, the animals were divided into three groups: PBS (n=4), VVLΔTKΔN1L (n=4), and VVL-TD-RFP (n=5). 1×10^7^ PFU of virus (VVLΔTKΔN1L or VVL-TD-RFP) or PBS was administered intraperitoneally on days 0, 2, and 4.

For intravenous delivery experiment, the DT6606 tumor model was established as described above. When the tumor volume reached 100 mm^3^, mice were treated with CAL-101 (10 mg/kg) or vehicle buffer by oral gavage followed 3 hours later by intravenous delivery with 1×10^8^ PFU of virus (VVL-TD-RFP or VVL-TD-mIL-27) or PBS at days 0, 2, 4, and 2×10^8^ PFU of virus or PBS at days 16, 18, and 20. Tumor growth was monitored using electronic calipers two times a week. The weight of the animals was recorded two times a week. When tumors reached 2,000 mm^3^ in volume or tumor ulceration occurred or animals lost 20% of their body weight, animals would be sacrificed. The tumor volume was calculated according to the following formula:


$$Tumor\;Volume=\frac{\pi w^{2}L}{6}$$

Where *w* is width, and L is length.

Tumor growth curves were terminated at the death of the first animal in each group, and individual tumor growth curve was terminated at the death of each animal, but survival in the group was monitored until the end of the experiment, and Kaplan-Meier survival plots were generated.

### Immune cell depletion

DT6606 subcutaneous tumors were established as described. When tumors reached 100 mm^3^, mice were stratified into five groups and received intratumoral injections of 1×10^8^ PFU of VVL-TD-mIL-27 or PBS in a total volume of 100 µL on days 0, 2, and 4. 1 day before commencement of viral treatment, 200 µg of anti-CD4 IgG (antibody clone GK1.5), anti-CD8 IgG (antibody clone TIB210), or anti-NK IgG (antibody clone PK136) (provided by Professor Shengdian Wang, the Chinese Academy of Sciences, Institute of Biophysics) was injected intraperitoneally in 200 µL PBS. Injections were continued two times a week for the duration of the experiment, and depletion was confirmed by flow cytometric analysis of splenocytes 48 hours after administration. Tumor growth was monitored two times a week. Other experimental methods are provided in online supplemental materials and methods.

### Statistical analysis

Cell flow cytometry data were analyzed using FlowJo_V.10 software. ImageJ was used to analyze the positive area of immunohistochemistry (IHC). GraphPad Prism V.9 was used for comparative statistical analysis. The results were represented as mean±SD. Dual condition comparisons were made using the unpaired Student’s *t*-test. For more than one condition, one-way or two-way analysis of variances were performed, with Tukey’s multiple comparison post-test to compare significance. Survival data were represented by Kaplan-Meier plots with log-rank analyses to delineate whether any differences between specific treatment pairs were statistically significant. P value<0.05 (*p<0.05, **p<0.01, ***p<0.001, and ****p<0.0001) was considered a statistically significant difference.

## Results

### *Vaccinia virus* VVL-TD-RFP replicates effectively in cancer cell lines and is cytotoxic *in vitro*

**Figure 1:**
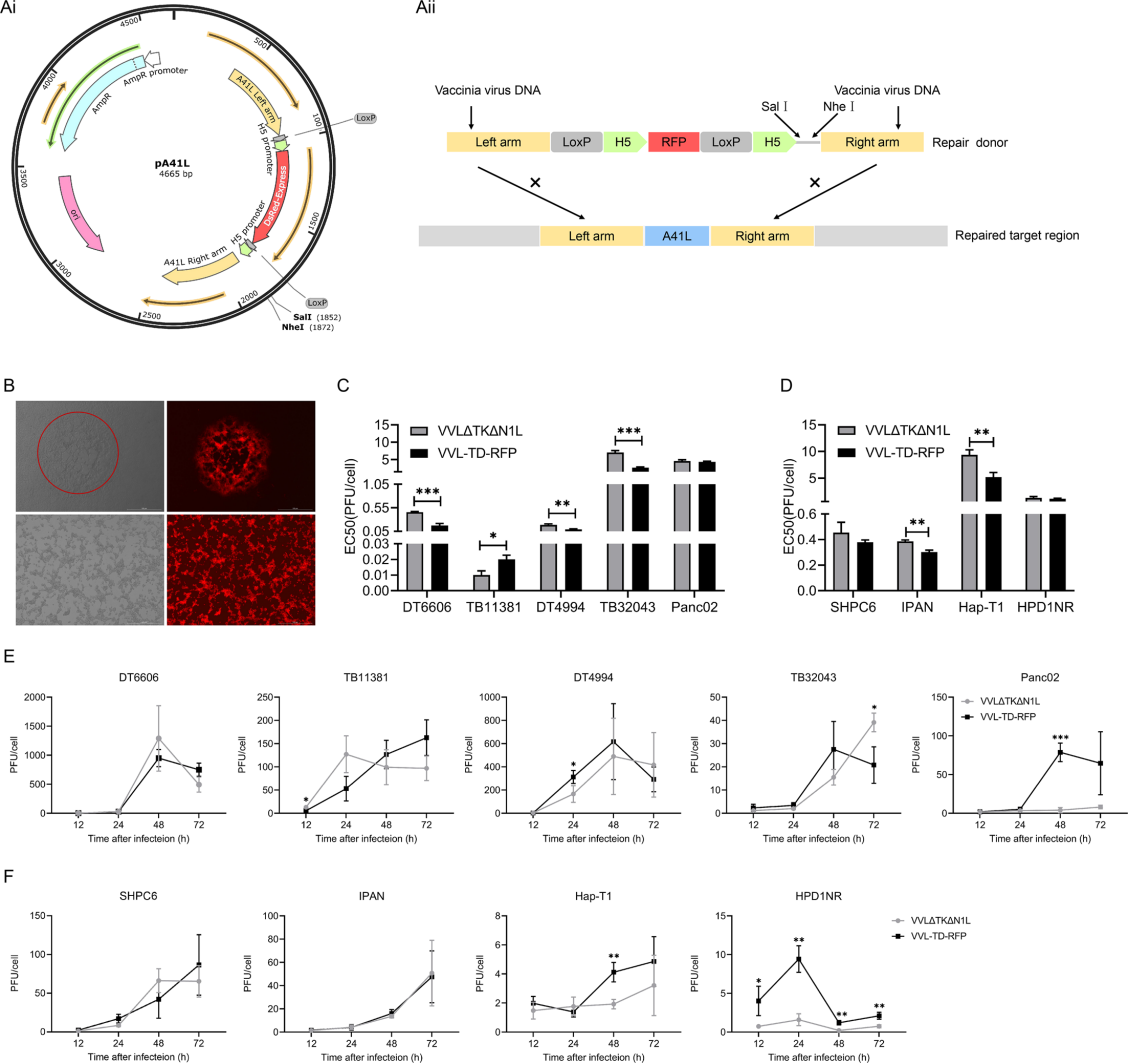
Construction of VVL-TD-RFP and its replication and cytotoxicity in PaCa cell lines *in vitro*. (**A**) Schematic diagram of virus construction. (**Ai**) Schematic diagram of pA41L plasmid. (**Aii**) Schematic of the homologous recombination cassette of the shuttle vector (repair donor vector) and repaired target region in the *vaccinia virus* genome. ×denotes homologous recombination. To generate VVL-TD-RFP, the shuttle plasmid pA41L was used for homologous recombination with VVLΔTKΔN1L by using the left and right flanking sequences of A41L. (**B**) The virus plaque formed in CV1 cells infected with VVL-TD-RFP (magnification×40). Mutant *vaccinia virus* was considered pure when all infected cells expressed the RFP marker (magnification×40). (**C, D**) Cytotoxicity of VVLΔTKΔN1L and VVL-TD-RFP against murine PaCa DT6606, TB11381, DT4994, TB32043, Panc02 cells (**C**), and Syrian hamster PaCa SHPC6, IPAN, Hap-T1, HPD1NR cells (**D**). Cell death was determined by the MTS assay 144 hours after infection. (**E, F**) Virus replication was determined in murine (**E**) and Syrian hamster (**F**) PaCa cell lines over 72 hours. Virus titers were determined using the TCID50 assay. In all cases, the mean±SD is shown, and Student’s unpaired *t*-test was used to assess significance at each time point (n=3). *p<0.05, **p<0.01, ***p<0.001. PaCa, pancreatic cancer; RFP, red fluorescent protein; VVL, *vaccinia virus* Lister strain; PFU, plaque forming unit.

Previously, we have described VVLΔTKΔN1L,^13^ which was used as a backbone to construct VVL-TD-RFP. CRISPR-Cas9-based homologous recombination was used to insert the red fluorescent protein (RFP) gene under the control of the H5 promoter into the A41L region of VVLΔTKΔN1L (figure 1A). VVL-TD-RFP exhibited red fluorescent plaques in CV1 cells (figure 1B). To determine the characteristics of VVL-TD-RFP *in vitro*, cytotoxicity and replication ability were detected in five murine and four Syrian hamster PaCa cell lines. The results showed that deletion of the A41L gene enhanced the cytotoxicity of VVL in DT6606, DT4994, TB32043, IPAN, and Hap-T1 cell lines, but moderately attenuated cytotoxicity in the TB11381 cell line. However, the overall cytotoxicity of VVL-TD-RFP was strongest in TB11381 compared with the other cell lines (figure 1C,D). These cell lines all support the replication of VVLΔTKΔN1L and VVL-TD-RFP. Compared with VVLΔTKΔN1L, the replication ability of VVL-TD-RFP was attenuated only in TB11381 cell line at hour 12 and in TB32043 cell line at hour 72, with enhanced or similar activity in other cell lines (figure 1E,F). These data indicated that the deletion of the A41L gene does not negatively affect the replication and cytotoxicity ability of VVL *in vitro*, and thus the A41L gene is non-essential for replication of VVL.

### VVL-TD-RFP improves tumor clearance and remodels the tumor microenvironment in pancreatic cancer subcutaneous tumor models

**Figure 2:**
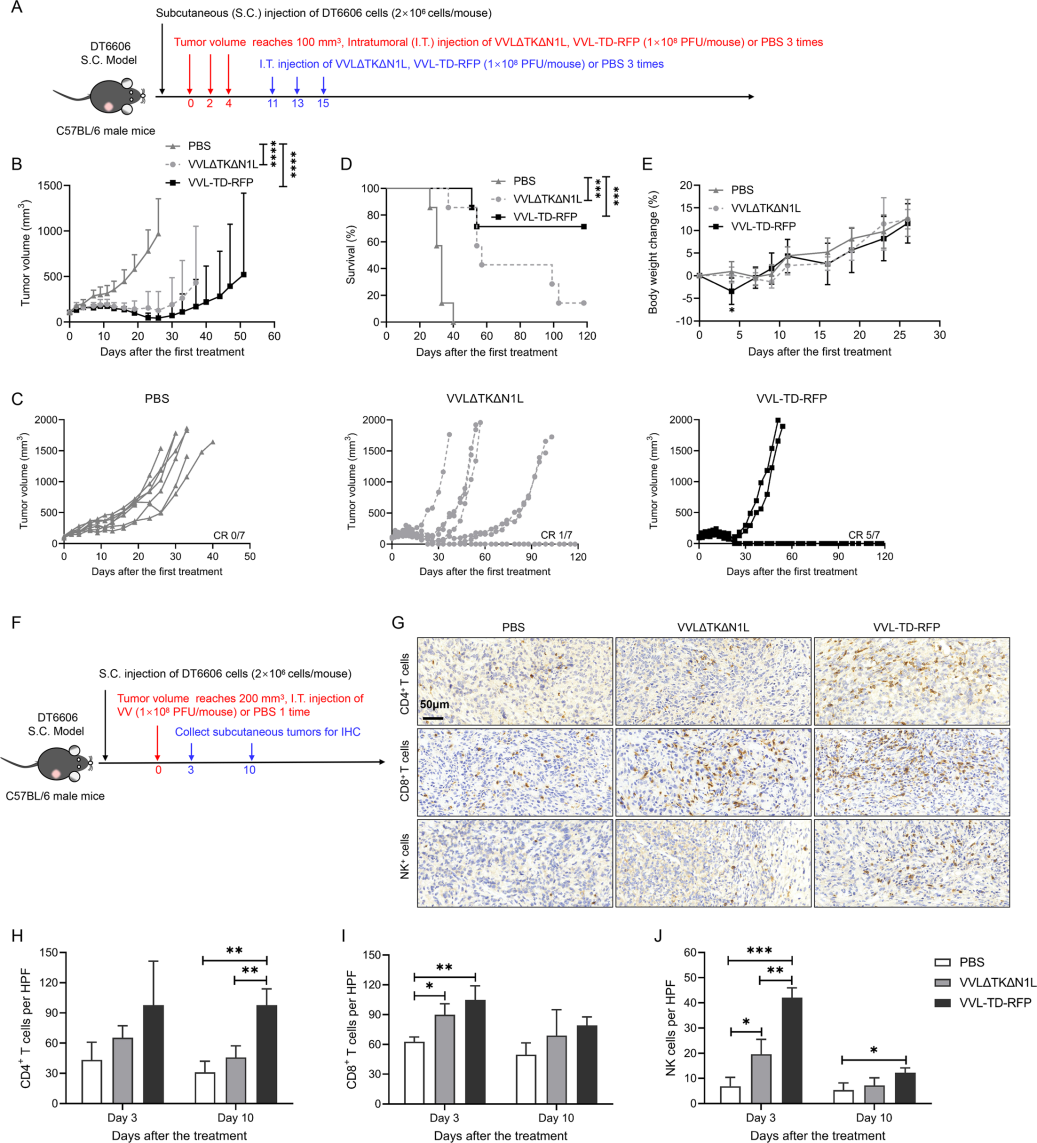
Intratumoral injection of VVL-TD-RFP improves tumor clearance and remodels the tumor microenvironment in a mouse pancreatic cancer subcutaneous tumor model. (**A**) Treatment schedule of the DT6606 subcutaneous (S.C.) tumor model. The DT6606 tumor models were established by inoculation of cells in the right flank of immune-competent male C57BL/6 mice (n=7/group). When the tumor volume reached 100 mm^3^, 1×10^8^ PFU of virus (VVLΔTKΔN1L or VVL-TD-RFP) or PBS was administered intratumorally (I.T.) on days 0, 2, 4, and 1×10^8^ PFU of virus (VVLΔTKΔN1L or VVL-TD-RFP) or PBS on days 11, 13, 15. (**B**) Mean tumor volume of mice. The mean±SD is shown. A two-way analysis of variance (ANOVA) with Tukey’s multiple comparison post-test was used to compare significance at all time points. Significance at day 26 is shown. (**C**) Individual tumor growth curve of mice. (**D**) A survival curve was plotted using Kaplan-Meier survival analysis with log-rank (Mantel-Cox) test. (**E**) Body weight change curve of mice. The mean±SD is shown. A two-way ANOVA with Tukey’s multiple comparison post-test was used to compare significance at all time points. Significance at day 4 is shown. (**F–J**) DT6606 pancreatic tumors were established subcutaneously in the flank of immune-competent C57BL/6 mice (n=6/group). When the tumor volume reached 200 mm^3^, mice were treated intratumorally once with 1×10^8^ PFU of virus (VVLΔTKΔN1L or VVL-TD-RFP) or PBS. Tumors were collected for IHC on days 3 and 10 after the first treatment to detect the infiltration of CD4^+^ T (**H**), CD8^+^ T (**I**), and NK (**J**) cells in the tumor tissues. Representative images of high magnification are shown (original magnification×400). CD4^+^ T cells display on day 10, CD8^+^ T and NK cells display on day 3. Cells per HPF were displayed graphically after 10 HPFs were counted. Mean cell number±SD is shown and a one-way ANOVA with Tukey’s multiple comparison post-test was used to assess significance (n=3/group). *p<0.05; **p<0.01; ***p<0.001; ****p<0.0001. IHC, immunohistochemistry; NK, natural killer cell; PBS, phosphate-buffered saline; RFP, red fluorescent protein; VVL, *vaccinia virus* Lister strain; PFU, plaque forming unit; HPF, high power field.

*In vivo*, the efficacy of VVL-TD-RFP was examined in the DT6606 subcutaneous tumor model in immunocompetent C57BL/6 mice (figure 2A). The results show that compared with the PBS group, the two viruses could significantly inhibit tumor growth (figure 2B). VVL-TD-RFP led to a higher complete response rate. After administration of VVL-TD-RFP, 71.4% of tumors were cleared, while in the VVLΔTKΔN1L group, only 14.3% of tumors were cleared. Neither group showed recurrence of cleared tumors and no tumors were cleared in the PBS group (figure 2C). Survival was significantly prolonged in both viral treatment groups compared with the PBS group (figure 2D), with VVL-TD-RFP non-significantly improving overall survival compared with VVLΔTKΔN1L. The body weight change curve showed that the VVL-TD-RFP group experienced a temporary decrease in body weight on the day of the second treatment compared with the PBS group, which may be due to the increase in virulence of VVL after deletion of the A41L gene (figure 2E). It has been reported that mice intranasally infected with the A41L-deleted VV WR strain lost more weight than those infected with wild-type virus,^17^ which is consistent with our results. We also investigated efficacy using the PaCa cell line TB13811 in a subcutaneous tumor model (online supplemental figure S1A). *In vitro*, the killing capacity of VVL-TD-RFP was attenuated compared with VVLΔTKΔN1L. However, *in vivo*, there was no statistical difference in the antitumor efficacy between the VVL-TD-RFP and the VVLΔTKΔN1L groups using this model (online supplemental figure S1B), and both virus treatments significantly prolonged survival (online supplemental figure S1C). Both viruses caused weight loss in mice during treatment, and the weight loss was within ethical limits (online supplemental figure S1D). The hamster PaCa cell line, SHPC6, in which the two viruses showed comparable killing efficiency, was used to create a Syrian hamster PaCa intraperitoneal dissemination model (online supplemental figure S1E). In this model, the progression of the tumor has been demonstrated to occur in a manner similar to end-stage human PaCa.^30^ Compared with VVLΔTKΔN1L treatment, intraperitoneal injection of VVL-TD-RFP treatment significantly prolonged animal survival (online supplemental figure S1F). Together, the A41L gene deletion increased the antitumor effect of oncolytic VV and appears to be an effective modification to the viral genome for the development of oncolytic virotherapy.

To determine the effect of VVL-TD-RFP on the TME of PaCa, we used IHC in the DT6606 subcutaneous tumor model to evaluate immune cell infiltration after a single intratumoral injection of the virus (figure 2F). The data indicate that at day 3 after the first treatment, the infiltration of CD8^+^ T cells and NK cells in the TME was significantly increased after treatment with both viruses compared with the PBS group. The infiltration of NK cells in the VVL-TD-RFP treated animals was more significant compared with the VVLΔTKΔN1L treated animals (figure 2I,J). At day 10 after the first treatment, VVL-TD-RFP treatment significantly increased tumor infiltration of CD4^+^ T cells, compared with both VVLΔTKΔN1L and PBS treatment (figure 2H). This data suggests that VVL-TD-RFP has a strong antitumor effect and can remodel the TME of PaCa.

### VVL-TD-RFP enhances innate and adaptive immune responses in mouse models of pancreatic cancer

**Figure 3:**
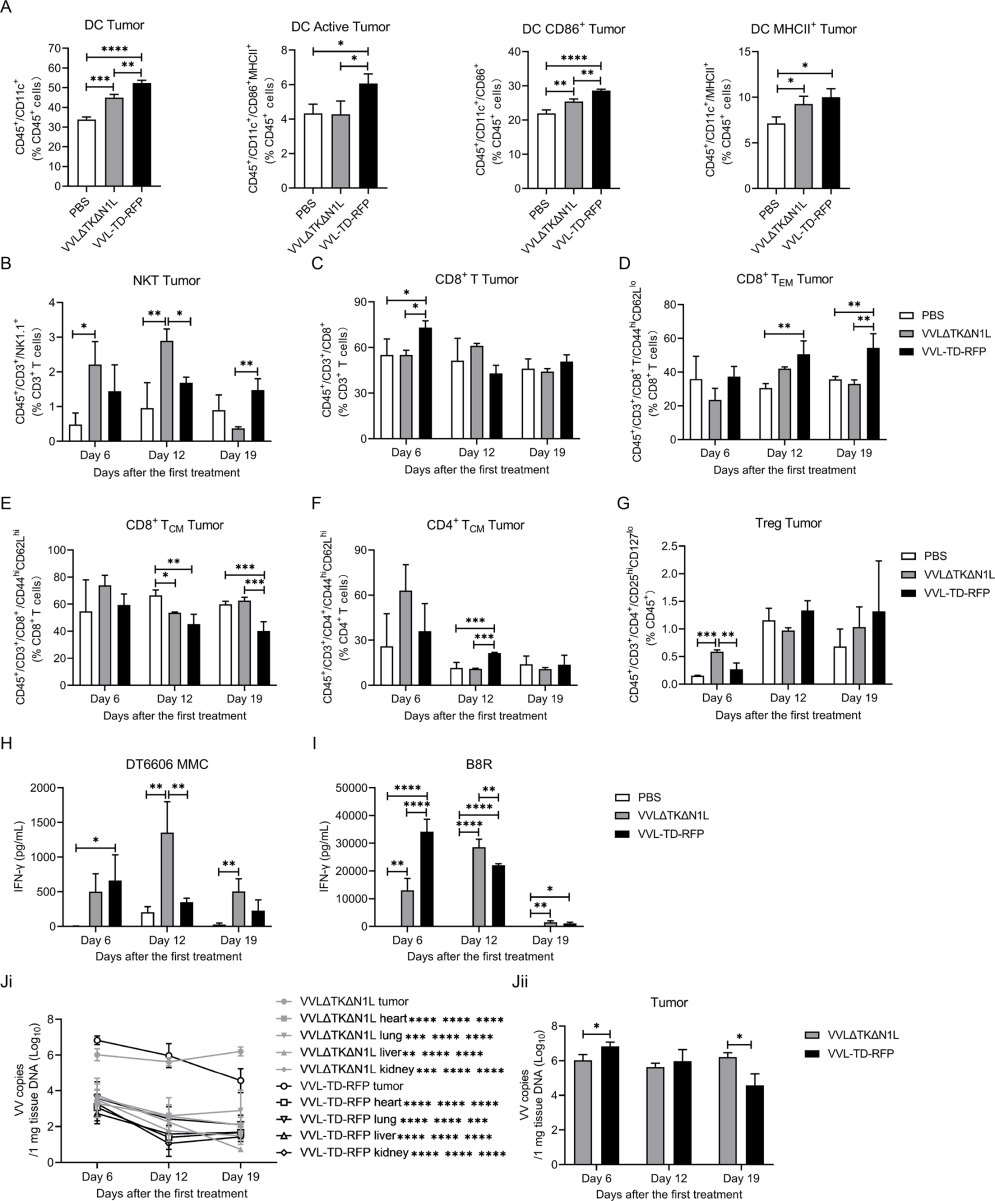
VVL-TD-RFP induces potent antitumor innate and adaptive immune responses after intratumoral administration. DT6606 tumor models were established by inoculation of cells in the right flank of C57BL/6 immune-competent male mice. When the tumor volume reached 230 mm^3^, mice were treated intratumorally with 1×10^8^ PFU of virus (VVLΔTKΔN1L, or VVL-TD-RFP) or PBS on days 0, 2, and 4 (n=10–11/group). (**A**) 6 days after the first treatment, tumors were collected and analyzed using flow cytometry (FC). DC was assessed by analyzing the CD11c^+^ population in the CD45^+^ population, activated DC was assessed by analyzing the CD86^+^MHC-II^+^ population in the CD45^+^ population, CD86^+^ DC was assessed by analyzing CD86^+^ population in the CD45^+^ population, MHC-II^+^ DC was assessed by analyzing MHC-II^+^ population in the CD45^+^ population. (**B–**G****), Tumors were collected and FC used to detect NK1.1^+^ population in the CD3^+^ population to analyze NKT cells (**B**), CD8^+^ population in the CD3^+^ population to analyze CD8^+^ T cells (**C**), CD44^hi^CD62L^lo^ population in the CD8^+^ population to analyze CD8^+^ TEM (**D**), CD44^hi^CD62L^hi^ population in the CD8^+^ population to analyze CD8^+^ TCM (**E**), CD44^hi^CD62L^hi^ population in the CD4^+^ population to analyze CD4^+^ TCM (**F**), CD25^hi^CD127^low^ population in the CD45^+^ population to analyze Treg cells (**G**) on days 6, 12, and 19 after the first treatment. (**H–I**), Splenocyte responses to MMC-treated DT6606 cells (**H**) or *vaccinia virus*-specific B8R peptide (**I**) were assayed using a restimulation assay on days 6, 12, and 19 after the first treatment. After 72 hours of incubation, IFN-γ production in the supernatant was determined using an ELISA. In all cases, the mean±SD is shown, and significance was assessed using a one-way ANOVA with Tukey’s multiple comparison post-test (n=3–4 mice/group/time point). *p<0.05; **p<0.01; ***p<0.001; ****p<0.0001. (**J**) Tumors, hearts, lungs, livers, and kidneys were collected, DNA was extracted, and viral load was analyzed using quantitative PCR shown as virus copy number per mg tissue DNA on days 6, 12, and 19 (n=3–4 mice/group/time point). The mean±SD is shown, and significance was analyzed using a two-way ANOVA with Tukey’s multiple comparisons post-test at each time point (**Ji**). The difference of each organ compared with tumor tissue is shown on days 6, 12, and 19. Student’s unpaired *t*-test was used to analyze the difference between the two viruses in tumor tissues (**Jii**). *p<0.05; **p<0.01; ***p<0.001; ****p<0.0001. Representative FC profiles are shown in online supplemental figure S3D. ANOVA, analysis of variance; DC, dendritic cell; IFN-γ, interferon γ; MHC, major histocompatibility complex; MMC, mitomycin C; NKT, natural killer T cell; PBS, phosphate-buffered saline; RFP, red fluorescent protein; TCM, central memory T cell; TEM, effector memory T cell; Treg, regulatory T cell; VVL, *vaccinia virus* Lister strain; PFU, plaque forming unit.

We evaluated the functional activity of VVL-TD-RFP using DT6606 subcutaneous tumors in male C57BL/6 mice (online supplemental figure S2A). We evaluated the immune cell populations in the tumor using flow cytometry (FC) with the gating strategies shown in online supplemental figure S2B,C. FC analysis of tumor innate immune cell populations indicated that at day 6 after the first treatment, both VVL-TD-RFP and VVLΔTKΔN1L treatments significantly increased the proportion of DC, CD86^+^ DC, and major histocompatibility complex (MHC)-II^+^ DC compared with PBS (figure 3A). Of note, VVL-TD-RFP treatment was more potent in increasing the proportion of DC, activated DC and CD86^+^ DC than VVLΔTKΔN1L treatment. Analysis of the natural killer T (NKT) cell population indicated that both viral treatments increased the proportion of NKT cells and that VVL-TD-RFP significantly increased the proportion of the NKT cell population compared with VVLΔTKΔN1L treatment at day 19 post-treatment (figure 3B). VVL-TD-RFP treatment did not modulate macrophage, M1-polarized macrophage (M1), or M2-polarized macrophage (M2) populations in the tumor tissues (online supplemental figure S3Ai–iii).

FC analysis of the tumor adaptive immune cell populations indicated that VVL-TD-RFP treatment enhanced the CD8^+^ T-cell population compared with VVLΔTKΔN1L and PBS treatments (figure 3C) and reduced the proportion of the Treg cells compared with VVLΔTKΔN1L treatment (figure 3G) at day 6 after the first treatment. At days 12 and 19, VVL-TD-RFP treatment significantly increased the proportion of CD8^+^ effector memory T cells (TEM), while the proportion of CD8^+^ central memory T cells (TCM) decreased significantly compared with VVLΔTKΔN1L and PBS treatments, indicating that VVL-TD-RFP promoted the transformation of CD8^+^ TCM to CD8^+^ TEM (figure 3D,E). On day 12, VVL-TD-RFP treatment elevated the CD4^+^ TCM cell population (figure 3F). Analysis of DC subpopulations and macrophage subpopulations in the spleen showed that VVL-TD-RFP treatment decreased the activated DC and MHC-II^+^ DC compared with PBS treatment (online supplemental figure S3Ci,ii), suggesting that both DC subpopulations are mobilized into tumor tissues to exert their antitumor effects. In addition, both virus treatments simultaneously decreased the proportion of macrophages and increased the proportion of M1 cells compared with PBS at day 6 post-treatment in the spleen (online supplemental figure S3Bi,ii), and VVL-TD-RFP treatment increased the proportion of M1 while decreasing the proportion of M2 cells compared with either PBS or VVL-TD-RFP treatment at day 19 post-treatment (online supplemental figure S3Bii,iii).

The results of the interferon (IFN)-γ release assay showed that both virus treatments were able to significantly increase the IFN-γ production by splenocytes stimulated *ex vivo* with mitomycin C (MMC)-treated DT6606 tumor cells. At day 6 after the first treatment, the VVL-TD-RFP treatment group significantly increased IFN-γ production compared with PBS (figure 3H). Stimulation with the VV-specific B8R peptide showed that IFN-γ production in splenocytes was significantly enhanced at days 6, 12, and 19 for both viruses, and VVL-TD-RFP induced a stronger antiviral immune response than VVLΔTKΔN1L at day 6. However, on day 19, the antiviral immune response induced by VVL-TD-RFP reduced, suggesting that there is an opportunity to re-administer viral therapy after day 19 (figure 3I).

To determine the distribution of the virus in organs and persistence in tumors after viral treatment, we analyzed the viral DNA load in tumors, hearts, lungs, livers, and kidneys by quantitative PCR detection. Viral copy number in tumors after treatment with both viruses was significantly higher than that in other organs at days 6, 12, and 19 after the first treatment (figure 3Ji). Interestingly, we found that the viral copy number in VVL-TD-RFP treated tumors was significantly higher than that of VVLΔTKΔN1L on day 6 after the first treatment (figure 3Jii), which is consistent with IFN-γ release after B8R stimulation. This may suggest that viral replication is stronger at this time and can lyse a large number of tumor cells to release more tumor-associated antigens, which is closely related to the significant increase of DC subsets in tumor tissues at the same time. However, at day 19, the viral copy number of the VVL-TD-RFP treatment group decreased significantly compared with the VVLΔTKΔN1L treatment (figure 3Jii), indicating that the deletion of the A41L gene increased the infiltration of immune cells in the TME, resulting in a quicker clearance of virus.

### Arming VVL-TD-RFP with IL-27 improves antitumor efficacy

IL-27 is a pleiotropic cytokine with potent antitumor activity that has been shown to have known effects on both adaptive and innate components of the immune system. To further remodel the immunosuppressive TME and to enhance the *vivo* efficacy of VVL-TD-RFP, the immunomodulatory cytokine IL-27 was integrated into the A41L region of the virus under the control of the H5 promoter, and the virus VVL-TD-mIL-27 (expressing murine IL-27) and VVL-TD-hIL-27 (expressing human IL-27) were successfully constructed (online supplemental figure S4Ai–iii). The results show both viruses retained replication activity and cytotoxicity in murine and Syrian hamster PaCa cell lines (online supplemental figure S4B–E), although a degree of attenuation compared with the parental virus was noted in some cell lines. Both viruses expressed IL-27 after infection of the murine (online supplemental figure S4F) and Syrian hamster (online supplemental figure S4G) cells *in vitro*.

**Figure 4:**
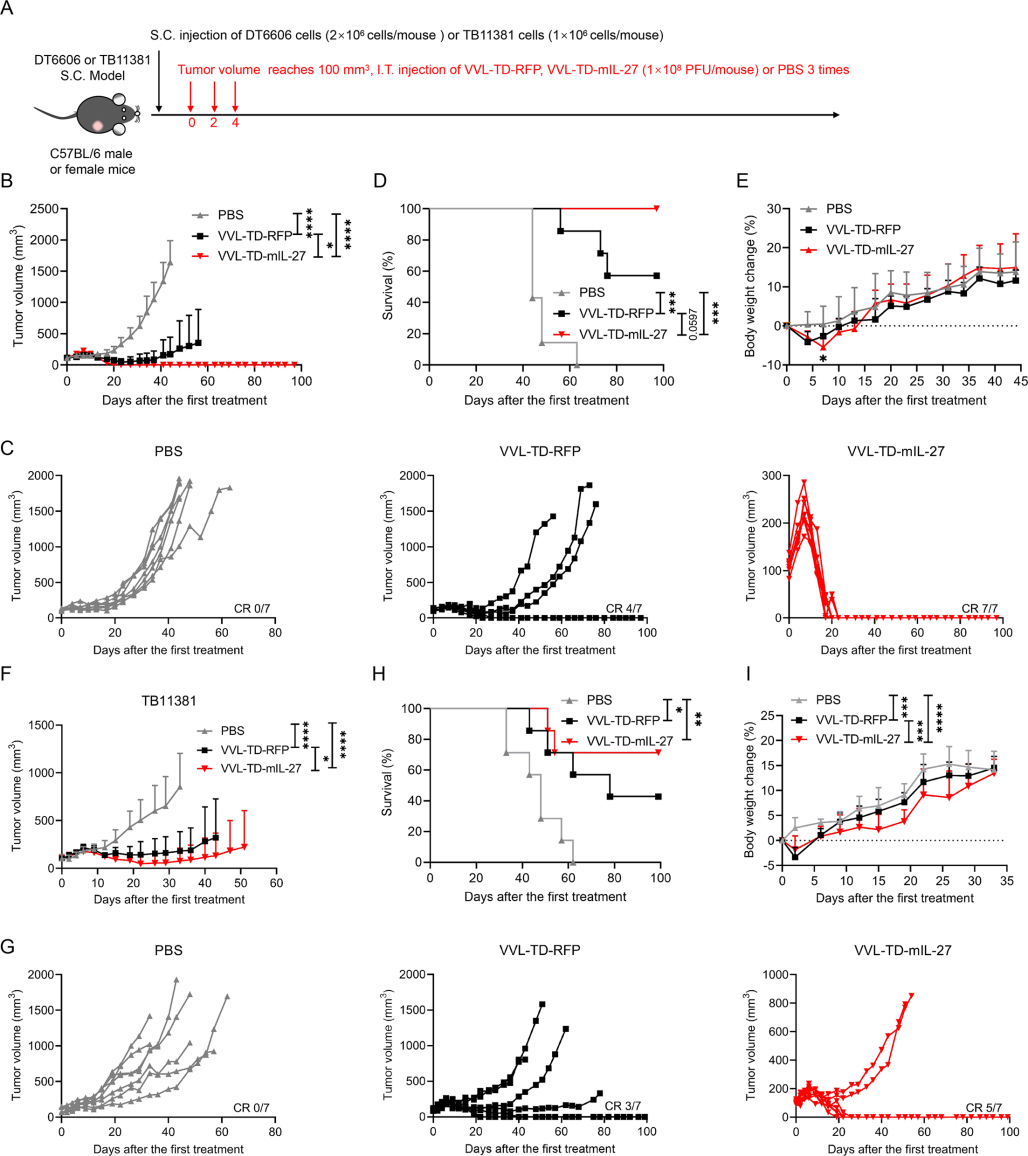
Arming VVL-TD-RFP with mIL-27 improves *in vivo* antitumor efficacy in mouse pancreatic cancer DT6606 and TB11381 subcutaneous tumor models. (**A**) Treatment schedule used in the DT6606 and TB11381 S.C. tumor models. The DT6606 and TB11381 tumor models were established by inoculation of cells in the right flank of C57BL/6 immune-competent male and female mice (n=7/group). When the tumor volume reached 100 mm^3^, the virus was delivered I.T. using 1×10^8^ PFU of virus or PBS on days 0, 2, and 4. (**B-E**) DT6606 S.C. tumor model. (**B**) Tumor growth curves are displayed until the first mouse in each group died. (**C**) Individual tumor growth curve of mice. (**D**) Kaplan-Meier survival analysis with log rank (Mantel-Cox) tests was used to assess survival. (**E**) Body weight change curve of mice. (**F–I**) TB11381 S.C. tumor model. (**F**) Tumor growth curves were displayed until the first mouse in each group died. (**G**) Individual tumor growth curve of mice. (**H**) Kaplan-Meier survival analysis with log rank (Mantel-Cox) test was used to assess survival. (**I**) Body weight change curve of mice. For all, mean±SD is shown. A two-way ANOVA with Tukey’s multiple comparison post-test was used to compare significance at all time points, and significance at (**B**) day 44 and (**E**) day 7 are shown. A two-way ANOVA with Tukey’s multiple comparison post-test was used to compare the significance between groups on day 33 (**F, I**). *p<0.05; **p<0.01; ***p<0.001; ****p<0.0001. ANOVA, analysis of variance; I.T., intratumorally; RFP, red fluorescent protein; S.C., subcutaneous; PBS, phosphate-buffered saline; VVL, *vaccinia virus* Lister strain; PFU, plaque forming unit.

*In vivo*, we first explored the optimal therapeutic dose of VVL-TD-mIL-27 in the DT6606 subcutaneous tumor model of PaCa, and the results showed that both the 5×10^7^ PFU and 1×10^8^ PFU dose groups resulted in 100% complete tumor remission (online supplemental figure S5A). The 1×10^8^ PFU dose group cleared all of the tumors by day 21, while the 5×10^7^ PFU dose group cleared tumors by day 55 (online supplemental figure S5B). Therefore, the 1×10^8^ PFU dose was used to evaluate the efficacy in DT6606 and TB11381 tumor models (figure 4A). After three intratumoral injections, VVL-TD-RFP and VVL-TD-mIL-27 treatments significantly inhibited tumor growth, but VVL-TD-mIL-27 exhibited significantly stronger antitumor effects compared with VVL-TD-RFP (figure 4B). VVL-TD-mIL-27 effected a 100% tumor cure rate compared with 57.1% for VVL-TD-RFP (figure 4C). Both viral treatments improved the survival rate of mice (figure 4D). The body weight change curve showed that although the body weight of the VVL-TD-mIL-27 group decreased temporarily compared with the PBS group on day 7, the weight loss was within the ethical limits and increased gradually after viral treatments (figure 4E).

We also investigated the efficacy of VVL-TD-mIL-27 using the murine PaCa TB11381 subcutaneous tumor model (figure 4F). VVL-TD-mIL-27 effected a 71.4% cure rate compared with a cure rate of 42.9% for VVL-TD-RFP (figure 4G), and both virus treatment groups improved survival compared with the PBS group (figure 4H). Using this model, we noted that mice in the VVL-TD-mIL-27 treatment group showed slower weight gain compared with the VVL-TD-RFP and PBS groups (figure 4I). This may be related to the recently reported function of IL-27 in the promotion of adipocyte thermogenesis and energy expenditure.^31^ Body weight in these animals, although lower compared with the control groups, did continue to increase as mice aged. These data showed that VVL-TD-mIL-27 is superior to VVL-TD-RFP in reducing tumor burden.

Previously, we demonstrated that CAL-101, a pharmacological inhibitor of PI3Kδ, inhibits phagocytosis of VV by macrophages and can promote the systemic delivery of VV.^32^ Here we found that oral delivery of CAL-101 followed by intravenous delivery of VVL-TD-mIL-27 was as effective as the intravenous delivery of VVL-TD-mIL-27 without CAL-101 support, and the tumor growth curves overlapped (online supplemental figure S5C,D). Both groups (+ or – CAL-101) retarded the growth of a few tumors compared with the PBS group and the CAL-101 combined with the VVL-TD-RFP group (online supplemental figure S5E). However, there was no difference in survival between the groups (online supplemental figure S5F). The weight change curve showed that weight gain in all three virus-treated groups was significantly slower compared with the PBS group. And, although the body weight decreased within ethical limits during treatment, it continued to increase after treatment, indicating that viral therapy was safe (online supplemental figure S5G).

To further verify the function of CAL-101 and whether IL-27 induces IL-10 expression, we detected the viral copy number and cytokine levels in the blood and tumor tissues at 8 and 72 hours after intravenous injection to the DT6606 subcutaneous tumor model (online supplemental figure S6A). The results showed that compared with the VVL-TD-mIL-27 group, the number of virus copies in blood and tumor tissues was significantly increased in the CAL-101 combined with VVL-TD-mIL-27 group at 8 and 72 hours after treatment (online supplemental figure S6Bi-Cii), which is consistent with our previous study showing that CAL-101 increased the intravenous delivery of VV.^32^ At hour 8 and 72 after treatment, mIL-27 levels in blood were significantly increased in the CAL-101 combined with VVL-TD-mIL-27 group compared with the CAL-101 combined with VVL-TD-RFP group (online supplemental figure S6Di,ii), but there was no difference in body weight changes between the two groups (online supplemental figure S5G), suggesting that intravenous treatment with VVL-TD-mIL-27 is safe. In addition, mIL-27 levels in tumor tissues were significantly increased in the CAL-101 combined with VVL-TD-mIL-27 group at 8 hours post-treatment, whereas mIL-27 levels in this group were significantly decreased at hour 72 post-treatment (online supplemental figure S6Ei,ii). Detection of the level of mIL-10 in tumor tissues showed that at 8 hours after treatment, the expression level of mIL-10 in the CAL-101 combined with VVL-TD-mIL-27 group tended to increase compared with the CAL-101 combined VVL-TD-RFP group, and at 72 hours after treatment the mIL-10 expression level in this group was significantly increased (online supplemental figure S6Fi,ii), indicating that IL-27 induced IL-10 production, which is consistent with previous results.^33^ Interestingly, IL-10 production delayed viral clearance from tumor tissues (online supplemental figure S6Ci,ii).

### VVL-TD-mIL-27 reduces Treg cells, promotes macrophage polarization to M1 phenotype, and exhibits potent anti-angiogenic activity

**Figure 5:**
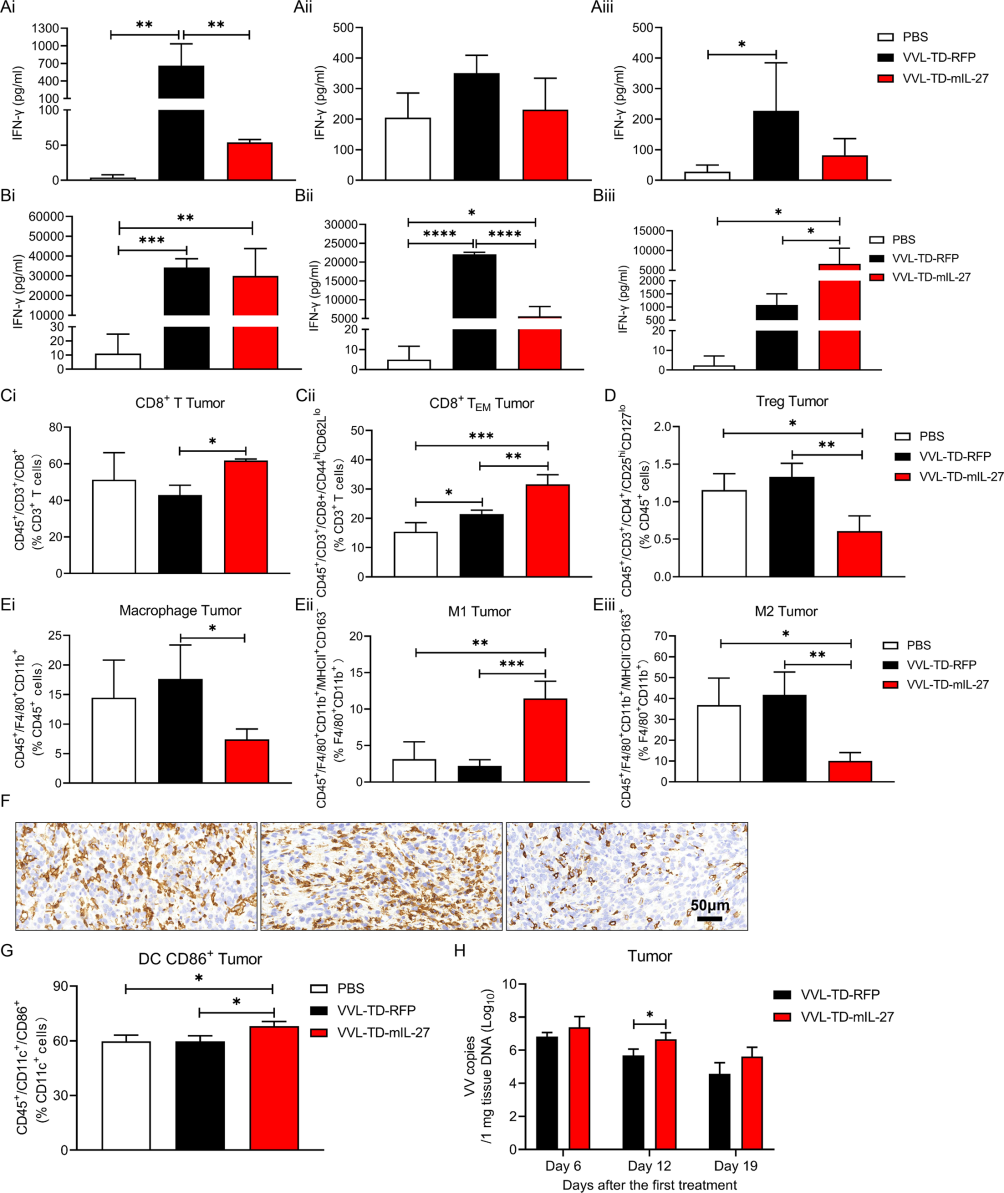
Analysis of IFN-γ expression, innate immune cells, T-cell populations, and viral persistence in tumors. C57BL/6 mice were subcutaneously inoculated with 3×10^6^ DT6606 cells. When the tumor volume reached 230 mm^3^ (day 0), virus was administered intratumorally using 1×10^8^ PFU or PBS on days 0, 2, and 4 (n=10–12/group). (**A–B**) Splenocyte responses to MMC-treated DT6606 cells (**A**) or viral proteins (B8R peptide) (**B**) were assayed using a restimulation assay on days 6 (**i**), 12 (**ii**), or 19 (**iii**) after the first treatment. After 72 hours of incubation, IFN-γ production in the supernatant was determined using an ELISA. (**C–E**) On day 12 after the first treatment, tumor tissues were collected for FC to detect CD8^+^ population in the CD3^+^ population to analyze CD8^+^ T cells (**Ci**), CD44^hi^CD62L^lo^ population in the CD3^+^ population to analyze CD8^+^ TEM (**Cii**), CD25^hi^CD127^low^ population in the CD45^+^ population to analyze Treg cells (**D**), F4/80^+^CD11b^+^ population in the CD45^+^ population to analyze macrophages (**Ei**), MHC-II^+^CD163^−^ population in the F4/80^+^CD11b^+^ population to analyze M1 (**Eii**), MHC-II^−^CD163^+^ population in the F4/80^+^CD11b^+^ population to analyze M2 (**Eiii**). (**F**) 12 days after the first treatment tumor sections were stained with F4/80 to assess macrophage infiltration (magnification ×400). (**G**) On day 12 after the first treatment, tumor tissues were collected for FC to detect CD86^+^ population in the CD11c^+^ population to analyze CD86^+^ DC. In the above cases, the mean±SD is shown, and significance was analyzed using a one-way ANOVA with Tukey’s multiple comparison post-test (n=3–4 mice/group/time point). *p<0.05; **p<0.01; ***p<0.001; ****p<0.0001. (**H**) Tumors were collected, DNA was extracted, and viral load was analyzed using quantitative PCR shown as virus copy number per mg tissue DNA on days 6, 12, and 19 (n=3–4 mice/group/time point). The mean±SD is shown. Significance was analyzed using a Student’s unpaired *t*-test at each time point. *p<0.05. Representative FC profiles are shown in online supplemental figure S9. ANOVA, analysis of variance; DC, dendritic cell; FC, flow cytometry; IFN-γ, interferon γ; MHC, major histocompatibility complex; MMC, mitomycin C; PBS, phosphate-buffered saline; RFP, red fluorescent protein; TEM, effector memory T cell; Treg, regulatory T cell; VVL, *vaccinia virus* Lister strain; PFU, plaque forming unit.

Given the strong antitumor capacity of VVL-TD-mIL-27 in the mouse PaCa DT6606 subcutaneous tumor model, we investigated the functional activity of this virus. *Ex vivo* restimulation assays demonstrated that after stimulation of splenocytes by MMC-treated DT6606 cells, no increase in IFN-γ release was observed in the VVL-TD-mIL-27 group, which was surprising given the improved antitumor efficacy provided by this virus (figure 5A). At all time points, after stimulation of splenocytes by B8R peptide, IFN-γ release was significantly increased in both viral groups, and IFN-γ release was significantly higher in the VVL-TD-mIL-27 group than in the other two groups at day 19 (figure 5B).

We analyzed tumor tissue innate and adaptive immune cell populations by FC on day 12 after treatment. Analysis of T-cell subsets showed that VVL-TD-mIL-27 treatment significantly increased the proportion of CD8^+^ T cells and CD8^+^ TEM compared with VVL-TD-RFP and PBS treatments (figure 5Ci,ii). In addition, VVL-TD-mIL-27 treatment significantly reduced the proportion of Treg cells (figure 5D). Analysis of innate immune cell populations showed that VVL-TD-mIL-27 treatment significantly decreased the proportion of macrophages compared with VVL-TD-RFP. Interestingly, VVL-TD-mIL-27 treatment resulted in a significant increase in the proportion of M1 and a significant decrease in the proportion of M2 compared with PBS and VVL-TD-RFP treatment (figure 5E,F). In addition, VVL-TD-mIL-27 treatment increased the proportion of CD86^+^ DC (figure 5G). VVL-TD-mIL-27 treatment did not further regulate DC, activated DC, and MHC-II^+^ DC (online supplemental figure S7A). Virus distribution experiments showed that the number of viral copies in tumor tissues was significantly higher than in the hearts, lungs, livers, and kidneys after VVL-TD-mIL-27 treatment (online supplemental figure S7B). On day 12 after the first treatment, more virus genomes were detected after VVL-TD-mIL-27 treatment compared with VVL-TD-RFP treatment (figure 5H) suggesting that mIL-27 can reduce viral clearance. This is consistent with the results of the IFN-γ release assay, which showed a weaker antiviral response in the VVL-TD-mIL-27 group at day 12 post-treatment. Analysis of serum IL-27 levels showed that compared with the VVL-TD-RFP group, the serum IL-27 level in the VVL-TD-mIL-27 group tended to increase on day 6 after treatment, returning to the base level by day 19 (online supplemental figure S7C).

**Figure 6:**
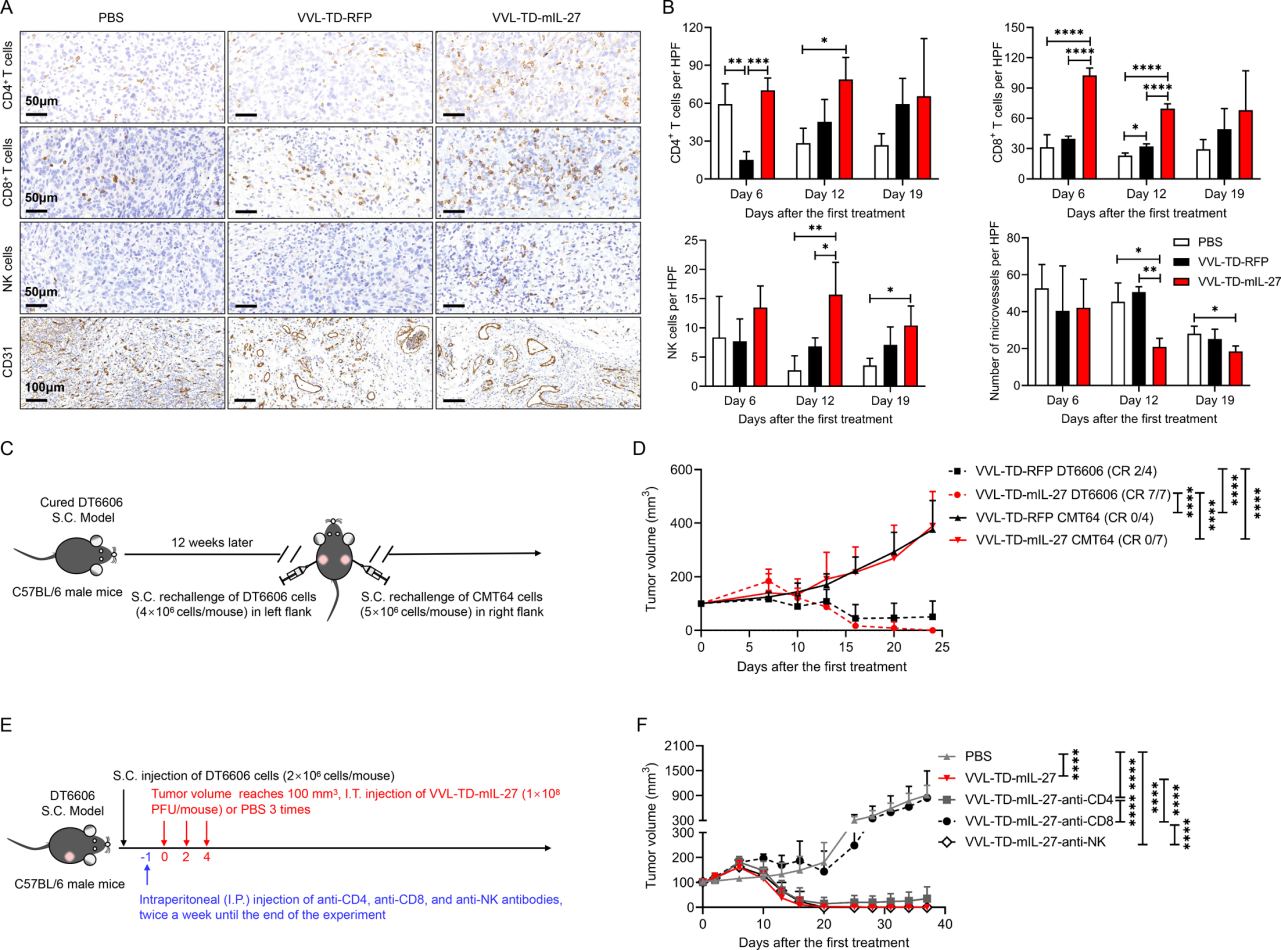
VVL-TD-mIL-27 treatment remodels pancreatic cancer tumor microenvironment, prompting the immune system to establish a specific antitumor immune response. The establishment of the tumor model and the treatment schedule were the same as in figure 5 (n=10–12/group). (**A–B**) Tumors were collected and stained for CD4^+^ T, CD8^+^ T, NK, and CD31 antibodies at days 6, 12, and 19 after the first treatment. Representative immunohistochemistry images are shown (CD4^+^ T, CD8^+^ T, and NK images magnification×400, CD31 images magnification×200). CD4^+^ T, CD8^+^ T, NK, and CD31 display on day 12 (**A**). Cells per HPF are shown graphically after 10 HPFs were counted (**B**). Notably, CD4^+^ T-cell staining included Treg cells, and NK staining included NK (CD3^−^NK1.1^+^) and NKT (CD3^+^NK1.1^+^). The mean±SD is shown, and significance was analyzed using a one-way ANOVA with Tukey’s multiple comparison post-test (n=3–4 mice/group/time point). *p<0.05; **p<0.01; ***p<0.001; ****p<0.0001. (**C–D**) In an efficacy experiment (figure 4B), 12 weeks after the tumors were cleared by I.T. treatment with VVL-TD-RFP or VVL-TD-mIL-27, the mice were rechallenged with 4×10^6^ DT6606 cells in the left flank and 5×10^6^ CMT64 cells in the right flank. VVL-TD-RFP n=4, VVL-TD-mIL-27 n=7. The mean tumor size±SD is shown and a two-way ANOVA with Tukey’s multiple comparison post-test was used to compare significance at day 24. ****p<0.0001. (**E–F**) DT6606 subcutaneous tumor model establishment and treatment schedule, cell-depleting antibodies commenced intraperitoneally in DT6606 tumor-bearing mice a day prior to the first I.T. treatment of the virus (n=7/group). The mean tumor size±SD is shown and a two-way ANOVA with Tukey’s multiple comparison post-test was used to compare significance at day 37. ****p<0.0001. ANOVA, analysis of variance; DC, dendritic cell; IFN-γ, interferon γ; I.T., intratumorally; NK, natural killer; PBS, phosphate-buffered saline; RFP, red fluorescent protein; S.C., subcutaneous; VVL, *vaccinia virus* Lister strain; PFU, plaque forming unit; HPF, high power field.

IHC results showed that VVL-TD-mIL-27 increased the infiltration of CD4^+^ T, CD8^+^ T, and NK cells in the TME at days 6 and 12 post-treatment compared with VVL-TD-RFP or PBS, and NK cells remained significantly increased at day 19. Next, we used the CD31 antibody to detect the effect of OV therapy on microvasculature in tumor tissues and found that VVL-TD-mIL-27 decreased the microvascular density in the TME compared with PBS and VVL-TD-RFP at days 12 and 19. Interestingly, the vasculatures in the TME were in a dilated state after treatment with VVL-TD-mIL-27 and VVL-TD-RFP, presumably because the OVs relieved the dense tumor tissue through oncolysis, thereby promoting normalization of the tumor vasculature (figure 6A,B). Additionally, VV coat protein was detected in tumor tissues following treatment with both VVL-TD-mIL-27 and VVL-TD-RFP over a defined time course (online supplemental figure S8). The results demonstrated that VVL-TD-mIL-27 treatment led to significantly higher VV protein expression within the tumor compared with the VVL-TD-RFP groups at days 6 and 12 post-treatment. This finding aligns with the viral copy number quantification (figure 5H), suggesting that VVL-TD-IL-27 enhances viral replication and persistence in tumors, thereby improving overall antitumor efficacy. In summary, IL-27 potentiates the antitumor effects of VVL-TD-RFP by increasing intratumoral viral replication, enhancing T-cell responses, promoting macrophage polarization toward the M1 phenotype, and exerting anti-angiogenic effects to remodel the TME.

### Treatment with VVL-TD-mIL-27 produces specific antitumor immune memory and long-term protection against tumor recurrence

The aim of oncolytic virotherapy is not only to eradicate the primary tumor but also to induce specific, long-term antitumor immunity to prevent tumor recurrence. 12 weeks after the primary tumors were cleared by viruses, the animals were rechallenged with 4×10^6^ DT6606 PaCa cells and 5×10^6^ lung cancer CMT64 cells (figure 6C). The results indicated that treatment with both viruses created long-term immunity to the DT6606 cells, resulting in rapid clearance of the PaCa cells, and the VVL-TD-mIL-27-treated animals cleared 100% of the secondary tumor compared with only 50% in the VVL-TD-RFP-treatment group at day 24 after inoculation. As expected, the subcutaneous tumors of an unrelated lung cancer cell line continued to grow in animals from both treatment groups (figure 6D).

To determine the contribution of different immune cell populations to therapeutic efficacy, CD4^+^ T, CD8^+^ T, and NK cells were depleted from mice before treatment of subcutaneous DT6606 tumors with VVL-TD-mIL-27 (figure 6E). FC was used to confirm immune subset depletion (online supplemental figure S10). The results showed that the depletion of CD8^+^ T cells had a significant detrimental effect on the therapeutic efficacy, the depletion of CD4^+^ T cells only modestly affected the therapeutic efficacy, and the virus maintained its antitumor effect when NK cells were depleted (figure 6F). The above results suggest that VVL-TD-mIL-27 is acting via CD8^+^ T cells to eliminate the tumor.

### Adaptive immune cell populations and innate immune cell populations are altered in lymph nodes and spleens after treatment with VVL-TD-mIL-27

**Figure 7:**
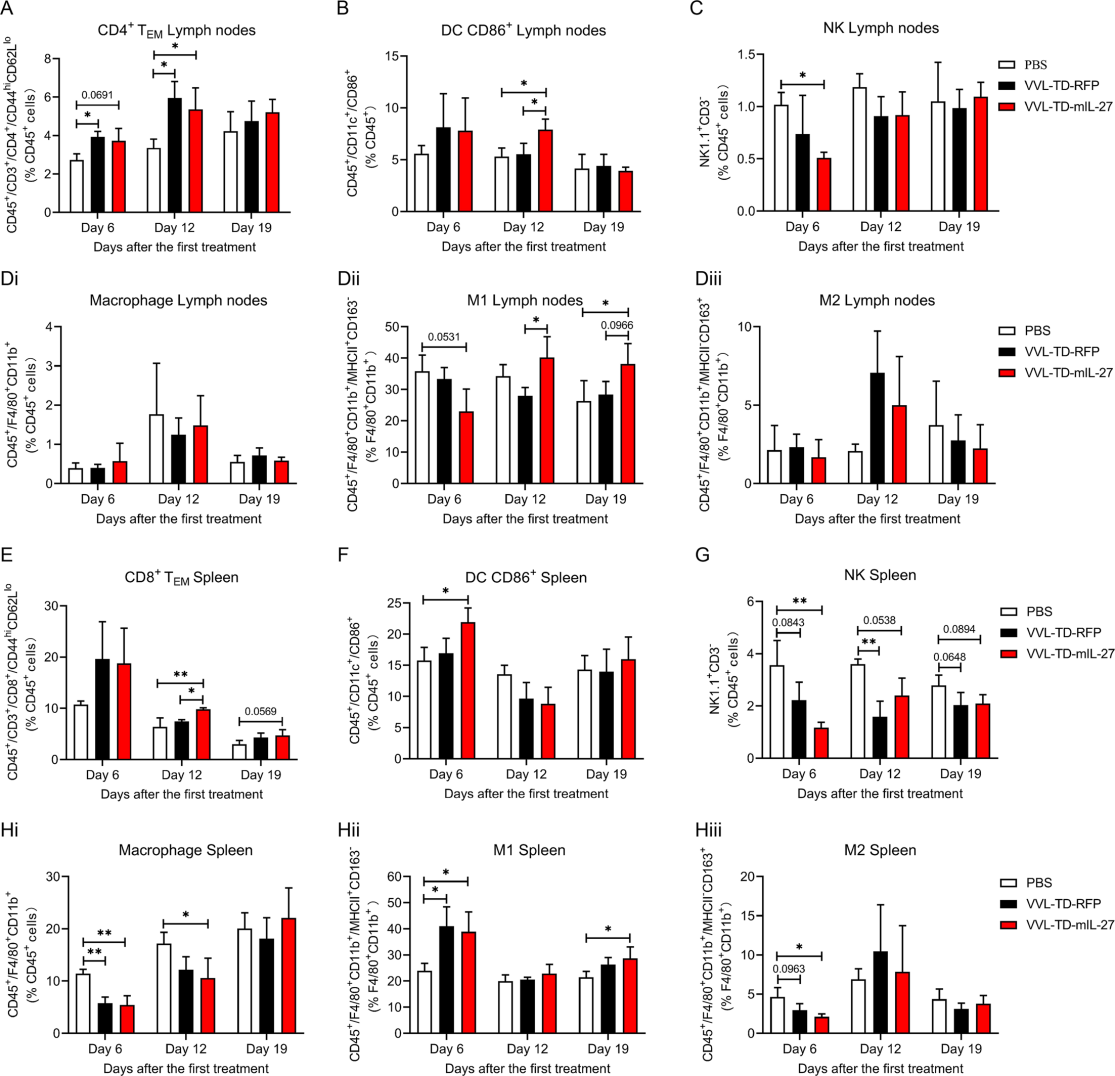
Analysis of adaptive and innate immune cell populations in lymph nodes and spleens of VVL-TD-RFP or VVL-TD-mIL-27 treated mice. DT6606 tumors were established and mice were treated intratumorally with VVL-TD-RFP or VVL-TD-mIL-27 following the same regimen as described for efficacy experiment. At days 6, 12, and 19, lymph nodes (**A–D**) and spleens (**E–H**) were harvested and analyzed using FC (n=3–4 mice/group/time point). (**A**) CD4^+^ TEM was assessed by analyzing the CD44^hi^CD62L^lo^ population in the CD45^+^ population. (**B**) CD86^+^ DC was assessed by analyzing the CD86^+^ population in the CD45^+^ population. (**C**) NK cells were assessed by analyzing NK1.1^+^CD3^- ^population in the CD45^+^ population. (**Di**) Macrophages were assessed by analyzing F4/80^+^CD11b^+^ population in the CD45^+^ population. (**Dii**) M1 was assessed by analyzing MHC-II^+^CD163^−^ population in the F4/80^+^CD11b^+^ population. (**Diii**) M2 was assessed by analyzing MHC-II^−^CD163^+^ population in the F4/80^+^CD11b^+^ population. (**E**) CD8^+^ TEM was assessed by analyzing CD44^hi^CD62L^lo^ population in the CD45^+^ population. (**F**) CD86^+^ DC was assessed by analyzing CD86^+^ population in the CD45^+^ population. (**G**) NK cells were assessed by analyzing NK1.1^+^CD3^-^ population in the CD45^+^ population. (**Hi**) Macrophages were assessed by analyzing F4/80^+^CD11b^+^ population in the CD45^+^ population. (**Hii**) M1 was assessed by analyzing MHC-II^+^CD163^−^ population in the F4/80^+^CD11b^+^ population. (**Hiii**) M2 was assessed by analyzing MHC-II^−^CD163^+^ population in the F4/80^+^CD11b^+^ population. In all cases, the mean±SD is shown, and significance was analyzed using a one-way analysis of variance with Tukey’s multiple comparison post-test (n=3–4 mice/group/time point). *p<0.05; **p<0.01. DC, dendritic cell; MHC, major histocompatibility complex; M1, M1-polarized macrophage; M2, M2-polarized macrophage; NK, natural killer; PBS, phosphate-buffered saline; RFP, red fluorescent protein; TEM, effector memory T cell; VVL, *vaccinia virus* Lister strain; PFU, plaque forming unit.

The draining lymph nodes play an important role in antitumor immunity, and the therapeutic efficacy of the virus involves the regulation of the immune system, thus we evaluated the lymph node and splenic immune cell populations in response to treatment. FC analysis of T-cell populations in draining lymph nodes showed that both VVL-TD-RFP treatment and VVL-TD-mIL-27 treatment increased the proportion of CD3^+^ T, and CD4^+^ T at day 6 (online supplemental figure S11A,D) and significantly increased the proportion of CD4^+^ TEM at day 12 compared with PBS (figure 7A). Analysis of innate immune cell populations showed that VVL-TD-mIL-27 treatment decreased the proportion of NK cells at day 6 (figure 7C), presumably being recruited to the TME. At day 12, VVL-TD-mIL-27 treatment increased the proportion of CD86^+^ DC cells compared with PBS treatment and VVL-TD-RFP treatment (figure 7B), and at days 12 and 19, VVL-TD-mIL-27 treatment increased the proportion of M1, compared with VVL-TD-RFP or PBS treatment (figure 7Dii). In addition, at day 19 both viral treatments decreased the proportion of CD8^+^ T cells (online supplemental figure S11B). There were no significant differences in CD8^+^ TEM, Treg, or NKT cells (online supplemental figure S11C,E,F). Macrophages and M2-polarized macrophages were also not significantly different among the three groups (figure 7Di,iii).

Analysis of T-cell populations of spleens showed that at day 12, VVL-TD-mIL-27 treatment increased the proportion of the CD8^+^ TEM population compared with VVL-TD-RFP treatment and PBS treatment (figure 7E). Analysis of innate immune cell populations showed that VVL-TD-mIL-27 treatment increased the proportion of CD86^+^ DC cells at day 6 (figure 7F), and VVL-TD-mIL-27 treatment decreased NK cells compared with PBS treatment at day 6, and a phenomenon also observed in the VVL-TD-RFP-treated group at day 12 (figure 7G), which is in contrast to the results observed in tumor tissues, likely due to the mobilization to the TME. Macrophage subsets changed mainly at early time points, both viral therapies reduced the proportion of macrophages (figure 7Hi) and increased the proportion of M1 (figure 7Hii), and VVL-TD-mIL-27 treatment decreased the proportion of the M2 population compared with PBS (figure 7Hiii). In addition, compared with PBS, VVL-TD-mIL-27 treatment decreased the macrophage population at day 12 (figure 7Hi), and an increase in the M1 population was still observed at day 19 (figure 7Hii). There was no significant difference in CD3^+^ T, CD8^+^ T, CD4^+^ T, CD4^+^ TEM, Treg, and NKT cell populations (online supplemental figure S11G–L).

## Discussion

OVs are widely acknowledged as powerful cancer immunotherapeutic agents that improve clinical outcomes by enhancing antitumor immunity in synergy with conventional therapies and other immunotherapies. VV have many inherent advantages that make them a promising agent for the treatment of PaCa.^8^ In particular, the VVL strain can replicate normally in hypoxic conditions and stably express exogenous genes, making it a promising vector for PaCa and potentially other hypoxic tumor types.^12^ We recently reported that the VVL strain with deletion of the TK and N1L genes (VVLΔTKΔN1L) has improved tumor specificity and remodeled the TME by enhancing innate and adaptive immune response.^13^

It is well known that around half of the VV genome encodes non-essential genes that affect virulence, host range, or interaction with the immune system.^34^ The A41L gene encodes a soluble immunomodulator similar to vCCI, which reduces immune cell infiltration and mediates profound anti-inflammatory effects *in vivo*.^15^ It has been reported that the VV WR strain and MVA strain with A41L gene deletion have enhanced immunogenicity and vaccine efficacy, and have been shown to induce enhanced CD8^+^ T-cell response.^17^ This deletion did not affect virus replication.^16^ Based on the above results, we speculate that the deletion of the A41L gene from VVL strain can enhance the immunogenicity of VV, increase the infiltration of immune cells in the TME, and activate an antitumor immune response, thus improving the therapeutic efficacy.

The results show that *in vitro*, A41L gene-deleted VV, VVL-TD-RFP retains the ability to replicate and kill tumor cells. In the mouse DT6606 subcutaneous tumor model of pancreatic ductal adenocarcinoma (PDAC), VVL-TD-RFP treatment resulted in a tumor clearance rate of 71.4%, compared with 14.3% of VVLΔTKΔN1L treatment. Further study showed that both viruses could induce CD8^+^ T cells to infiltrate into tumor tissues, but VVL-TD-RFP significantly increased the infiltration of CD4^+^ T and NK cells in the TME. We speculate that deletion of the A41L gene removes the inhibition of chemokines, thereby allowing the recruitment of multiple immune cells into the TME. Consistent with our results, the VV WR strain with A41L gene deletion was injected subcutaneously into rabbits and resulted in increased CD43^+^ leukocyte infiltration in the infected area.^16^

DCs are powerful and specialized antigen-presenting cells that play an important role in bridging innate and adaptive immunity and are the key determinants in initiating and maintaining an effective T cell-mediated antitumor immune response.^35^ It has been reported that conventional DCs are reduced in pancreatic tumors and have lower levels of co-stimulatory molecules and maturation markers leading to poorer antigen-presenting function compared with those in lung cancer. Furthermore, overcoming conventional DC defects in early PDAC leads to disease suppression, and restoration of conventional DC function in advanced PDAC restores tumor-suppressive immunity.^36^ Previously, our study showed that VVLΔTK∆N1L infection enhanced overall DC activation.^13^ Interestingly, here, we observed that both viruses increased DCs, and the expression of the co-stimulatory molecule CD86 on DCs in tumor tissues on day 6 after the first treatment, and VVL-TD-RFP significantly enhanced this response. At the same time point, we detected more copies of VVL-TD-RFP in tumor tissues, suggesting that VVL-TD-RFP replicated more strongly so that more tumor cells are lysed and a large number of tumor-associated antigens are released to promote DCs infiltration and activation. Meanwhile, we found that at day 6, the proportion of CD8^+^ T cells increased in the tumor tissues. On days 12 and 19, the proportion of CD8^+^ TEM in the tumor tissues increased significantly. This indicates that DC initiates and maintains effective CD8^+^ T cell-mediated antitumor immune response, which is the core of effective antitumor immune response. This is consistent with previous reports that deletion of the A41L gene in the WR strain and MVA strain enhanced the CD8^+^ T-cell immune response.^17^

Preclinical and clinical data suggest that the effective way to enhance the antitumor immune response induced by OVT platforms is to arm viral vectors with immuno-stimulatory transgenes. The IL-12 family includes four heterodimeric cytokines: IL-12, IL-27, IL-23, and IL-35, of which IL-35 is a potent inhibitory cytokine.^37^ IL-12 is regarded as one of the most effective activators of the immune system and has a strong antitumor effect, but excessive toxicity has limited its clinical application.^18^ IL-27, like IL-12, plays an important role in innate and adaptive immunity, both of which promote T helper 1 cell differentiation and CD 8^+^ T and NK cell responses.^19^ It has been reported that IL-23 enhances the infiltration of M2-polarized macrophages and neutrophils in the TME and promotes angiogenesis.^38^ In contrast, IL-27 promotes macrophage polarization to the M1 phenotype,^24^ and inhibits angiogenesis.^23^ In addition, the anti-inflammatory effect of IL-27 primarily induces IL-10 production.^33^ Interestingly, our previous studies showed that IL-10 increases VV persistence, thus maximizing the oncolytic effect and antitumor immunity associated with VV.^39^ Here, we armed our VV with IL-27 and the results show that arming with IL-27 further enhances the antitumor effect of VVL-TD-RFP, leading to 100% tumor clearance and a long-term specific antitumor immune response in the DT6606 subcutaneous tumor model.

Further studies demonstrated that IL-27 beneficially remodeled the TME of PDAC. Analysis of adaptive immune cell populations showed that VVL-TD-mIL-27 treatment significantly increased the proportion of CD8^+^ TEM in tumor tissues, and this effect was also observed in spleens at day 12, suggesting that the immune response induced by viral therapy extended from the tumor local to the whole body. *In vivo* experiments showed that the depletion of CD8^+^ T cells eliminated the antitumor effects associated with VVL-TD-mIL-27. Consistent with our results, it has been reported that IL-27 can enhance tumor-specific CD8^+^ T-cell expansion, program the memory T-cell differentiation of CD8^+^ T cells and increase CD8^+^ TEM production.^40^ Treg cells inhibit the aberrant immune response against self-antigens as well as the antitumor immune response, and the infiltration of a large number of Treg cells into tumor tissues is associated with poor prognosis.^41^ In this study, we demonstrated that VVL-TD-mIL-27 treatment significantly reduced the Treg cell population in the TME. It was recently reported that AAV expressing IL-27 enhanced antitumor efficacy by inducing Treg cells depletion.^26^

In addition to enhancing the adaptive antitumor immune response, VVL-TD-mIL-27 also enhanced the innate immune response. Macrophages are crucial in the innate immune system and are a major component of the mononuclear phagocytic system,^42^ which is a key component of the TME and is largely associated with poor prognosis.^43^ Macrophages are highly plastic, with antitumor M1 and tumor-promoting M2 populations representing the extremes of a continuum in different states.^44^ M2-polarized macrophages predominate in the TME and promote tumor progression by inhibiting the antitumor immune response and promoting angiogenesis, tumor cell proliferation, and metastasis.^45^ Thus, M2-polarized macrophages in the TME are a strong prognostic indicator of reduced overall survival in many malignancies.^46^ Our results showed that treatment with VVL-TD-mIL-27 reduced macrophages in the TME and that M1 predominated in these cells, while the proportion of M2 was significantly decreased, suggesting that IL-27 promoted M2 to M1 polarization. IL-27 has been reported to exert antitumor effects by promoting the differentiation of hematopoietic stem cells toward M1 macrophages,^47^ and also to improve the efficacy of PaCa treatment by targeting M2-polarized TAM.^24^ In addition, IL-27 has also been reported to promote the expression of the co-stimulatory molecule CD86.^48^ In the present study, we reconfirmed this idea by showing a significant increase in the proportion of CD86^+^ DC cells in tumor tissues after VVL-TD-mIL-27 treatment at day 6, and this phenomenon was also observed in lymph nodes and spleens. In addition, we found that VVL-TD-mIL-27 treatment significantly increased the infiltration of NK cells in the TME. Consistent with our results, AAV-IL-27 could enhance the accumulation of NK cells in tumor tissues after intratumoral injection.^49^ We also noted that IL-27 has an anti-angiogenic effect and VVL-TD-mIL-27 treatment reduced the microvascular density in the TME, in line with previous reports of the anti-angiogenic effect of IL-27 in tumors.^23^ Of note, we found in our studies that after stimulation of splenocytes by MMC-treated DT6606 cells, the release of IFN-γ in the VVL-TD-mIL-27 group did not increase. It is possible that the amount of antigen in MMC-treated DT6606 cells was insufficient to induce a response *ex vivo*, but interestingly, it has been reported that during OV treatment, the enhanced antiviral immune response improves the antitumor effect via recognition of viral antigens on infected tumor cells.^50^

IL-27 can induce the production of the anti-inflammatory cytokine IL-10,^33^ and our previous studies found that IL-10 could inhibit VV clearance to enhance antitumor effects.^39^ We have also previously reported that pretreatment of immunocompetent mice with CAL-101, a selective inhibitor of PI3 kinase-δ, prior to intravenous delivery of oncolytic VV transiently prevented the uptake of the virus by macrophages, thereby significantly improving virus delivery to tumors.^32^ Here, we again confirmed that the copy number of virus in blood and tumor tissues was significantly increased after CAL-101 treatment. We found that CAL-101 treatment resulted in a significant increase in the viral copy number in the tumor tissues at day 12 post-treatment, suggesting that IL-27 can delay the clearance of VVs during intravenous delivery by inducing the production of IL-10, thus allowing more viruses to reach the tumor tissue to exert antitumor effects. However, there was no difference in antitumor efficacy between the CAL-101 combined with VVL-TD-mIL-27 group and the VVL-TD-mIL-27 group, which may be due to the fact that, OV therapy exerts antitumor effects through multiple mechanisms, and although the increase in viral copy number in tumor tissues enhanced the oncolysis, the IL-27-armed virus also elicited a strong antiviral immune response. These warrant further investigation of the underlying mechanism.

Altogether, these results demonstrated that VVL-TD-IL-27 is a potential cancer immunotherapy agent that can achieve a complete response in all tumors by remodeling the TME of PDAC and inducing a long-term, specific antitumor immune response.

## Supplementary material

10.1136/jitc-2024-010341 online supplemental file 1

10.1136/jitc-2024-010341 online supplemental file 2

## Data Availability

Data sharing not applicable as no datasets generated and/or analysed for this study.
