# Supplementary material for: Novel oncolytic vaccinia virus armed with interleukin-27 is a potential therapeutic agent for the treatment of murine pancreatic cancer
Source: J Immunother Cancer. 2025 May 11;13(5):e010341. doi: 10.1136/jitc-2024-010341 (PMC12067774; doi:10.1136/jitc-2024-010341)
Supplement: online supplemental file 1 [file jitc-13-5-s001.pdf]

A novel oncolytic vaccinia virus armed with Interleukin-27 is a potential therapeutic agent for the treatment of murine pancreatic cancer

Yangyang Jia,[1] Yanru Wang,[1] Guanghao Zhao,[1] Yong Yang,[1] Wenyi Yan,[1] Ruimin Wang,[1] Bing Han,[1] Lihong Wang,[2] Zhe Zhang,[3] Lijuan Chen,[4] Nicholas R Lemoine,[1,5] Louisa S Chard Dunmall,[5] Pengju Wang*,[1] Yaohe Wang*[1,5]

Supplementary Materials and Methods

Cell lines

The murine pancreatic ductal adenocarcinoma (PDAC) cell line DT6606 and the preinvasive pancreatic cancer (PanIN) cell line DT4994 were cultured from LSL-KRAS$^{G12D/+}$; Pdx-1-Cre mice that had developed PDAC.[1] The PDAC cell line TB11381 was cultured from LSL-KRAS$^{G12D/+}$; Trp53R172H/+; Pdx-1-Cre mice that had developed PDAC.[2] These three cell lines were kindly provided by David Tuveson (Cancer Research UK Cambridge Research Institute, Cambridge, United Kingdom; now at Cold Spring Harbor Laboratory). The murine PDAC cell lines TB32043, Panc02, and lung cancer cell line CMT64 were obtained from CRUK, Clare Hall, Herts, UK. CV1 (African monkey kidney) cells were obtained from the American Type Culture Collection (ATCC). The Syrian hamster PDAC cell lines SHPC6 and IPAN were kindly provided by W. Wold (St. Louis University, St. Louis, MO, USA), and the HPD1NR and Hap-T1 cell lines were purchased from the German Collection of Microorganisms and Cell Cultures. DT6606, DT4994, TB32043, TB11381, Panc02, CMT64, SHPC6, IPAN, Hap-T1, and CV1 cells were cultured in Dulbecco's modified Eagle's medium (DMEM) supplemented with 10% fetal bovine serum (FBS). The Syrian hamster PDAC cell line HPD1NR was cultured in RPMI 1640 supplemented with 10% FBS. All cells were cultured in an incubator at 37℃ and 5% CO_2.

Vaccinia virus replication assay

Pancreatic cancer cell lines were seeded at 2×10^5 cells per well in 6-well plates in triplicate and infected with VVLΔTKΔN1L, VVL-TD-RFP, or VVL-TD-IL-27 16 hours later at a multiplicity of infection (MOI) of 1 plaque-forming unit (PFU) per cell. Cells and supernatants were collected at 12, 24, 48, and 72 hours after infection, and titers were determined by TCID50. Viral burst titers were converted to PFU per cell based on the number of cells present at viral infection.

Cell cytotoxicity assay

Pancreatic cancer cells were seeded at 2×10^3 cells per well in 96-well plates in triplicate and infected with VVLΔTKΔN1L, VVL-TD-RFP, or VVL-TD-IL-27 16 hours later at a starting MOI of 1,000 PFU per cell. Six days after infection, an MTS non-radioactive cell proliferation assay kit (Promega) was used according to the

manufacturer's instructions. Cell viability was detected by measuring absorbance at 490 nm using a 96-well plate absorbance reader (SpectraMax Absorbance Reader CMax Plus) and a dose-response curve created by non-linear regression allowing determination of an EC50 value (dose required to kill 50% of cells) as previously described.[3]

Enzyme-linked immunosorbent assay (ELISA)

IL-27, IL-10, and interferon γ (IFN-γ) protein levels were quantified using Human IL-27 DuoSet ELISA (R&D Systems, DY2526-05), Mouse IL-27 Uncoated ELISA Kit (Invitrogen, 88-7274-86), Mouse IL-10 Uncoated ELISA Kit (Invitrogen, 88-7105-88), or Mouse IFN gamma Uncoated ELISA Kit (Invitrogen, 88-7314-77) according to the manufacturers' instructions. Where appropriate, data were normalized to the number of cells at the time of infection.

Functional studies

2×10^6 DT6606 cells were injected subcutaneously into the right flank of 6-7 weeks immune-competent male C57BL/6 mice. When the tumors reached a volume of 200 mm^3, mice were randomly divided into 3 groups, and each mouse was intratumorally injected once with 1×10^8 PFU/100 µL VVLΔTKΔN1L or VVL-TD-RFP for the treatment group, and 100 µL of PBS for the control group. On days 3 and 10 after the treatments, tumor tissues were collected for IHC (3 mice per group per time point).

3×10^6 DT6606 cells were injected subcutaneously into the right flank of 6-7 weeks immune-competent male C57BL/6 mice. When the tumor volume reached 230 mm^3, mice were randomly divided into 4 groups and intratumorally injected with 1×10^8 PFU/100 µL of VVLΔTKΔN1L, VVL-TD-RFP or VVL-TD-mIL-27 for the treatment group, and 100 µL of PBS for the control group on days 0, 2, and 4. On days 6, 12, and 19 after the first treatment, the subcutaneous tumors, spleens, draining lymph nodes, hearts, lungs, livers, kidneys, and serum were collected for further investigation (3-4 mice per group per time point), including flow cytometry (FC) analysis, IHC, IFN-γ release assay, real-time quantitative PCR, and ELISA.

2×10^6 DT6606 cells were implanted subcutaneously into the right flank of 6-7 weeks immune-competent male C57BL/6 mice (n=6/group). When the tumor volume reached 140 mm^3, mice were treated with CAL-101 (10 mg/kg) or vehicle buffer by oral gavage followed 3 hours later with 1×10^8 PFU VVL-TD-RFP or VVL-TD-mIL-27 via I.V. injection once. At 8 and 72 hours after treatment, blood and tumor tissues were collected, and the virus copy number was detected by qPCR, and the levels of mIL-27 and mIL-10 were detected by ELISA (3 mice per group per time point).

Rechallenge of tumor-free animals

Subcutaneous tumors in the VVL-TD-RFP (n=4) or VVL-TD-mIL-27 (n=7) groups were cured in C57BL/6 mice. 12 weeks after the tumors were cleared, 4×10^6 DT6606 cells (twice the number of cells used to establish the tumor model) were inoculated subcutaneously on the opposite side to the site of primary tumor injection, and 5×10^6 CMT64 (the same number of cells used to establish the subcutaneous tumor

model) were subcutaneously inoculated on the same side as the primary tumor. Tumor growth was monitored.

Preparation of splenocytes

Spleens were extracted from mice, placed in complete T cell media (RPMI medium 1640, 10% FBS, 1% streptomycin/penicillin, 1% sodium pyruvate), and flushed through 70 µm cell strainers to prepare single cells. Red blood cells (RBCs) were lysed using RBC Lysis Buffer (Solarbio, R1010), the samples washed in PBS and resuspended in a complete T cell medium.

IFN-γ release assay of spleen cells

Splenocytes were seeded into 96-well plates in triplicate at 5×10^5 cells/well/100 µL. Re-stimulation was performed with 5×10^4 DT6606 cells treated with mitomycin C (MMC) (MedChemExpress, HY-13316) or VV-specific B8R peptide (10µg) (TSYKFESV) (GL Biochem) in a volume of 100 µL. The restimulated splenocytes were incubated at 37℃ with 5% CO_2 for 3 days and the supernatant was collected for ELISA to detect the concentration of IFN-γ.

Real-time quantitative PCR

Tumors, hearts, lungs, livers, kidneys and blood were harvested and homogenized. DNA was extracted using the Vazyme FastPure Cell/Tissue DNA Isolation Mini Kit (Vazyme, DC102). The primers and probes used were designed to target the VV late transcription factor 1 (VLTF-1) gene:
Forward:5'-*AACCATAGAAGCCAACGAATCC*-3',
Reverse:5'-*TGAGACATACAAGGGTGGTGAAGT*-3',
Probe: *ATTTTAGAACAGAAATACCC*.[4]
Primers were provided by Sangon Biotech. qPCR was carried out using the ABI STEPONE PLUS system and the Premix Ex Taq™ (Probe qPCR) (Takara, RR390A).

Tumor cells and lymphocytes preparation

Tumor tissues were homogenized and incubated with 1× collagenase Ⅰ (Sigma, C0130), collagenase Ⅳ (Sigma, C5138), and hyaluronidase for 30 min at 37℃ on a shaker. The lymph nodes were gently ground, the tumor cells and lymphocytes were separated with a 70 µm strainer, and the cells were suspended in T cell medium.

Flow cytometry analysis of immune cell populations

Tumor cells, lymphocytes and splenocytes were stained with Zombie Yellow™ Fixable Viability Kit (Biolegend, #423103), CD45 (PerCP) (Biolegend, #103130), CD3 (BV510) (Biolegend, #100234), CD4 (FITC) (Biolegend, #100406), CD8a (APC-Cy7) (Biolegend, #100714), CD25 (PE) (Biolegend, #102008), CD127 (APC) (Biolegend, #135012), CD62L (PE-cy7) (Biolegend, #104418), CD44 (BV785) (Biolegend, #103059), NK1.1 (BV421) (Biolegend, #108732), CD11b (PE-cy7) (Biolegend, #101216), APC (F4/80) (Biolegend, #123116), CD163 (PE) (Biolegend, #155308), CD11c (BV785) (Biolegend, #117336), I-A/I-E (MHCⅡ) (FITC) (Biolegend, #107606), CD86 (BV605) (Biolegend,

#105125) at 4℃ for 30 minutes in the dark. Cells were fixed with FluoroFix Buffer (Biolegend, #422101) for 60 minutes, washed with PBS and analyzed on an ACEA NovoCyte flow cytometer. Data were analyzed using FlowJo software (Tree Star, Inc.).

Immunohistochemistry

Tissue sections were baked, dehydrated, and subjected to antigen retrieval under high pressure in pH 6.0 citrate buffer, followed by incubation with 3% hydrogen peroxide for 25 min at room temperature (RT) to block endogenous peroxidase activity. Sections were then incubated overnight at 4°C with primary antibodies against CD4 (1:100, #25229S), CD8α (1:200, #98941S), NKR-P1C (1:100, Abcam, #ab289542), F4/80 (1:200, #70076S), CD31 (1:100, #77699S), and *Vaccinia virus* (1:1,000, BIO-RAD, #9503-2057). After secondary antibody incubation (50 min, RT), DAB development (5 min), and hematoxylin counterstaining (3 min), sections were dehydrated in anhydrous ethanol and mounted. For quantification, CD4/CD8/NK-stained samples were analyzed in 10 random high-power fields (HPF, ×400) per sample for positive cell counts, while CD31-stained samples were assessed in 7 HPFs (×200), with each distinct CD31-positive endothelial cell or cluster counted as a microvascular unit.[5] In addition, greater than 80% of the area was selected (×200) and the positive area with VV protein expression was counted using ImageJ.

Supplementary Materials and Methods References

1. Hingorani SR, Petricoin EF, Maitra A, et al. Preinvasive and invasive ductal pancreatic cancer and its early detection in the mouse. *Cancer Cell* 2003;4(6):437-50. doi: 10.1016/s1535-6108(03)00309-x
2. Hingorani SR, Wang L, Multani AS, et al. Trp53R172H and KrasG12D cooperate to promote chromosomal instability and widely metastatic pancreatic ductal adenocarcinoma in mice. *Cancer Cell* 2005;7(5):469-83. doi: 10.1016/j.ccr.2005.04.023
3. Wang Y, Hallden G, Hill R, et al. E3 gene manipulations affect oncolytic adenovirus activity in immunocompetent tumor models. *Nat Biotechnol* 2003;21(11):1328-35. doi: 10.1038/nbt887 [published Online First: 20031012]
4. Chard LS, Maniati E, Wang P, et al. A vaccinia virus armed with interleukin-10 is a promising therapeutic agent for treatment of murine pancreatic cancer. *Clin Cancer Res* 2015;21(2):405-16. doi: 10.1158/1078-0432.Ccr-14-0464 [published Online First: 20141121]
5. Martin DK, Uckermann O, Bertram A, et al. Differential growth inhibition of cerebral metastases by anti-angiogenic compounds. Anticancer Res 2014;34(7):3293-302.
