# Supplementary material for: Novel oncolytic vaccinia virus armed with interleukin-27 is a potential therapeutic agent for the treatment of murine pancreatic cancer
Source: J Immunother Cancer. 2025 May 11;13(5):e010341. doi: 10.1136/jitc-2024-010341 (PMC12067774; doi:10.1136/jitc-2024-010341)
Supplement: online supplemental file 2 [file jitc-13-5-s002.pdf]

A novel oncolytic vaccinia virus armed with Interleukin-27 is a potential therapeutic agent for the treatment of murine pancreatic cancer

Yangyang Jia,[1] Yanru Wang,[1] Guanghao Zhao,[1] Yong Yang,[1] Wenyi Yan,[1] Ruimin Wang,[1] Bing Han,[1] Lihong Wang,[2] Zhe Zhang,[3] Lijuan Chen,[4] Nicholas R Lemoine,[1,5] Louisa S Chard Dunmall,[5] Pengju Wang*,[1] Yaohe Wang*[1,5]

Supplementary Figures

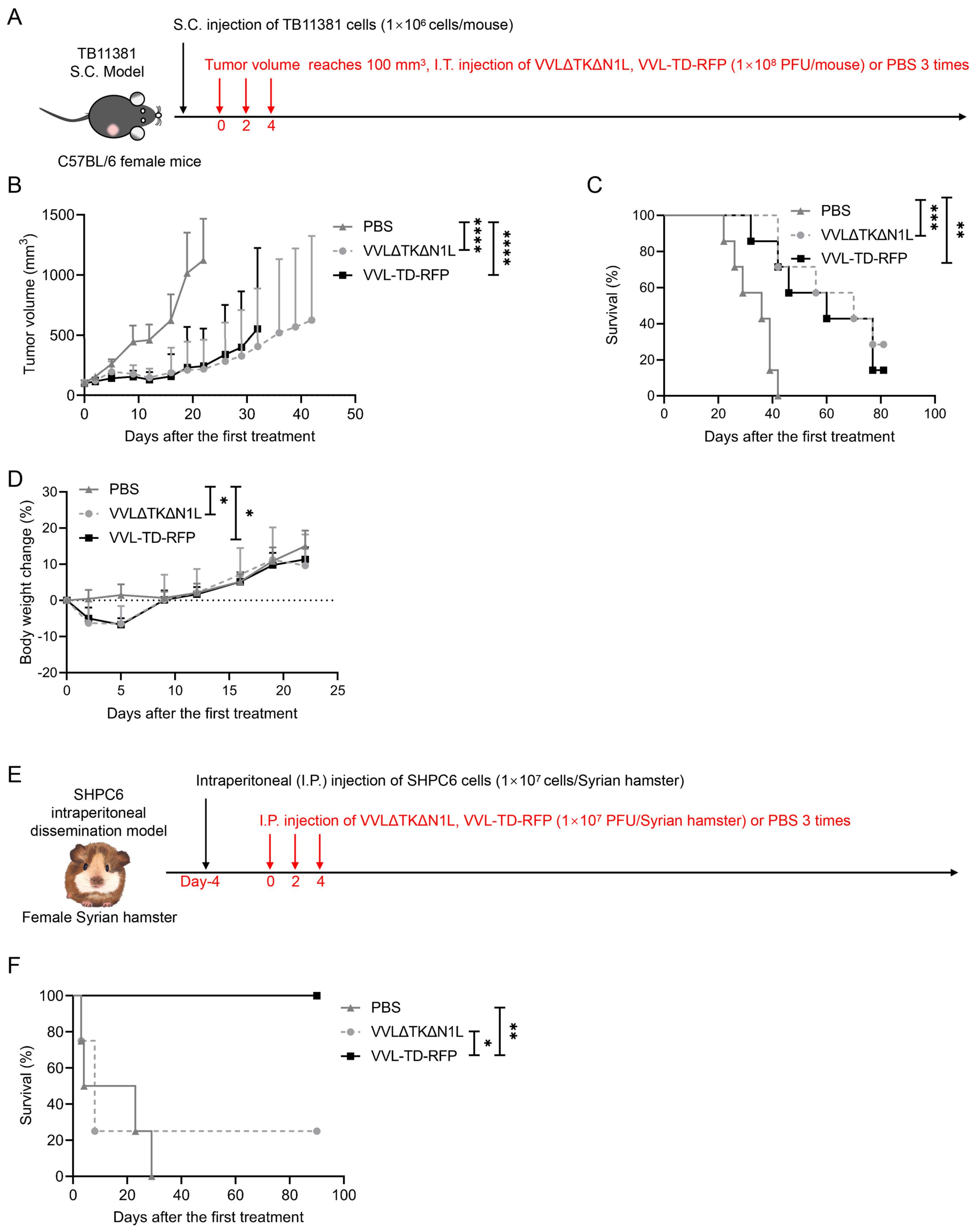

Supplementary figure 1 Evaluating the antitumor efficacy of VVL-TD-RFP in the TB11381 subcutaneous model and the SHPC6 intraperitoneal dissemination model. (**A**) Treatment schedule of the TB11381 subcutaneous (S.C.) tumor model. TB11381 cells were subcutaneously inoculated into the right axilla of immune-competent female C57BL/6 mice (n=7/group). When the tumor volume reached 100 mm^3, 1×10^8 PFU of virus (VVLΔTKΔN1L or VVL-TD-RFP) or PBS was administered intratumorally on days 0, 2, and 4. (**B**) Mean tumor volume of mice. (**C**) A survival curve was plotted using Kaplan-Meier survival analysis with log rank (Mantel-Cox) test. (**D**) Body weight change curve of mice. A two-way ANOVA with Tukey's multiple comparison post-test was used to compare the significance between groups on day 22 (**B, D**). *P<0.05; **p<0.01; ***p<0.001; ****p<0.0001. (**E**) Treatment schedule of the SHPC6 intraperitoneal (I.P.) dissemination model. SHPC6 cells were inoculated into the lower right peritoneal cavity of Syrian hamsters. Four days later, the animals were divided into 3 groups PBS (n=4), VVLΔTKΔN1L (n=4), and VVL-TD-RFP (n=5). 1×10^7 PFU of virus (VVLΔTKΔN1L or VVL-TD-RFP) or PBS was administered intraperitoneally (I.P.) on days 0, 2, and 4. (**F**) A survival curve was plotted using Kaplan-Meier survival analysis with log rank (Mantel-Cox) test. *P<0.05; **p<0.01. PBS, phosphate-buffered saline; VVL, *vaccinia virus* Lister strain; PFU, plaque forming unit; S.C., subcutaneous; I.T., intratumoral; I.P., intraperitoneal.

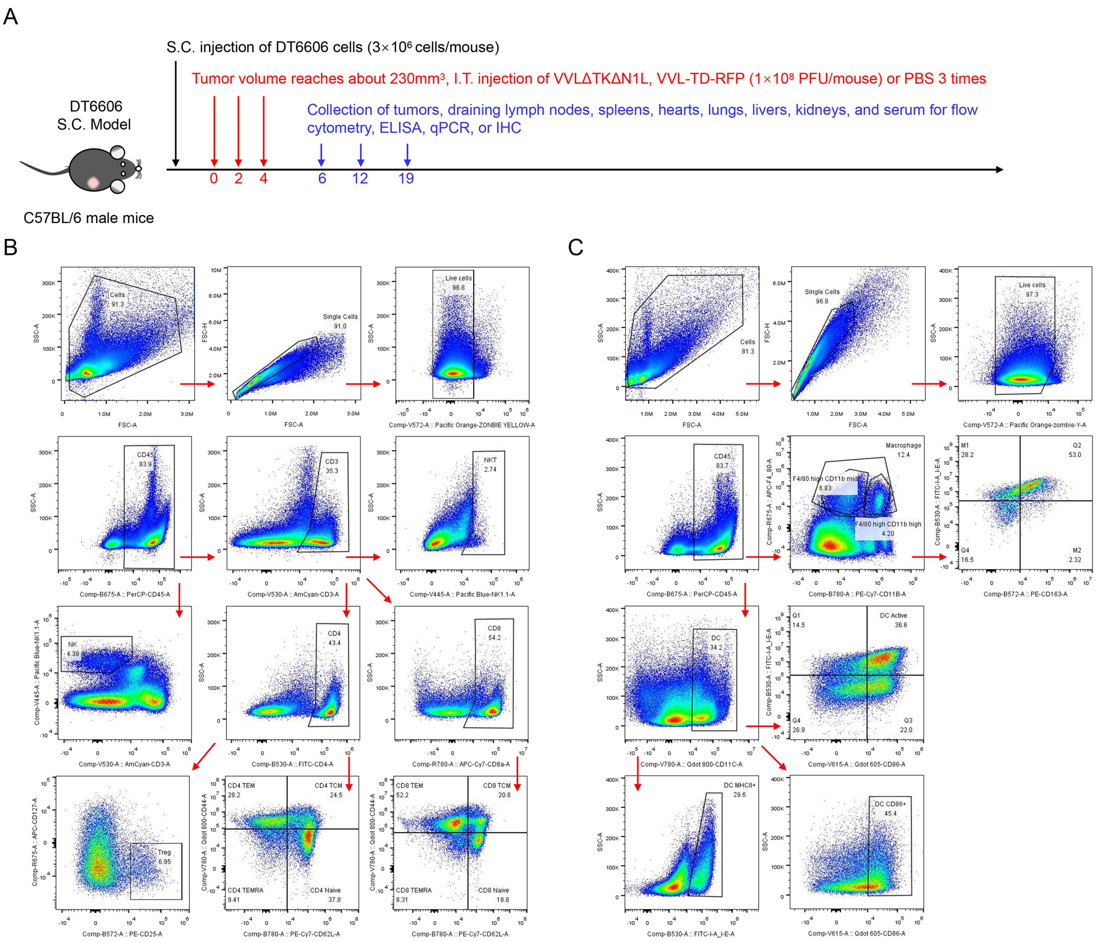

Supplementary figure 2 Schematic of the experimental protocol for the study of antitumor mechanisms and flow cytometry (FC) gating strategy. (**A**) The DT6606 tumor models were established by inoculation of cells in the right flank of C57BL/6 immune-competent male mice. When the tumor volume reached 230 mm^3, mice were treated intratumorally (I.T.) with 1×10^8 PFU VVLΔTKΔN1L, VVL-TD-RFP or PBS on days 0, 2, and 4 (n=10-11/group). On days 6, 12, and 19 after the first treatment, tumors, draining lymph nodes, spleens, hearts, lungs, livers, kidneys, and serum were collected for FC, ELISA, qPCR, or IHC (3-4 mice/group/time point). (**B**) T cell, NK, and NKT cell populations FC gating strategies. (**C**) Dendritic cell (DC) and macrophage populations gating strategies. PBS, phosphate-buffered saline; VVL, *vaccinia virus* Lister strain; PFU, plaque forming unit; S.C., subcutaneous; I.T., intratumoral; NK, natural killer cell; NKT, natural killer T cell; DC, dendritic cell; MHC, major histocompatibility complex; M1, M1-polarized macrophage; M2, M2-polarized macrophage; Treg, regulatory T cell; TEM, effector memory T cell; TCM, central memory T cell.

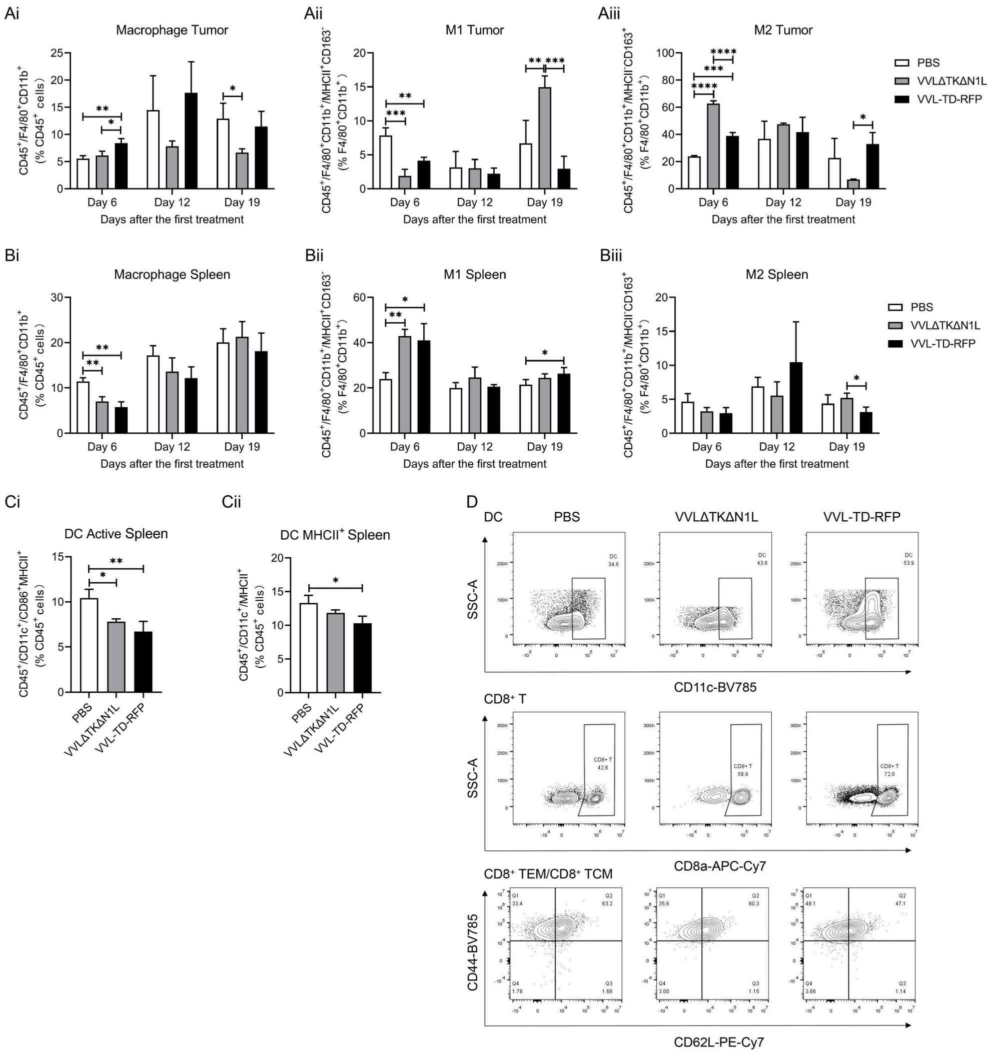

Supplementary figure 3 Analysis of immune cell populations in tumors and spleens. The establishment of the tumor model and the treatment schedule were the same as described in Figure 3. (**A-B**) Macrophage subsets were analyzed in tumor tissues and spleens by FC on days 6, 12, and 19 after the first treatment. (**Ai, Bi**) Macrophages were assessed by analyzing F4/80$^+$CD11b$^+$ population in the CD45$^+$ population. (**Aii, Bii**) M1 was assessed by analyzing MHCⅡ$^+$CD163$^-$ population in the F4/80$^+$CD11b$^+$ population. (**Aiii, Biii**) M2 was assessed by analyzing MHCⅡ$^-$CD163$^+$ population in the F4/80$^+$CD11b$^+$ population. (**C**) DC subsets were analyzed in spleens by FC at days 6 after the first treatment. Activated DC was assessed by analyzing CD86$^+$MHCⅡ$^+$ population in the CD45$^+$ population (C**i**), MHCⅡ$^+$ DC was assessed by analyzing MHCⅡ$^+$ population in the CD45$^+$ population (C**ii**). (**D**) Representative diagram of FC analysis of DC, CD8$^+$ T, CD8$^+$ TEM, CD8$^+$ TCM cells in the tumor tissues. In all cases, the mean ± SD is shown (n=3-4 mice/group/time point) and significance was analyzed using a one-way ANOVA with Tukey's multiple comparison post-test. *P<0.05; **p<0.01; ***p<0.001; ****p<0.0001. PBS, phosphate-buffered saline; VVL, *vaccinia virus* Lister strain; M1, M1-polarized macrophage; M2, M2-polarized macrophage; DC, Dendritic cell; MHC, major histocompatibility complex; TEM, effector memory T cell; TCM, central memory T cell.

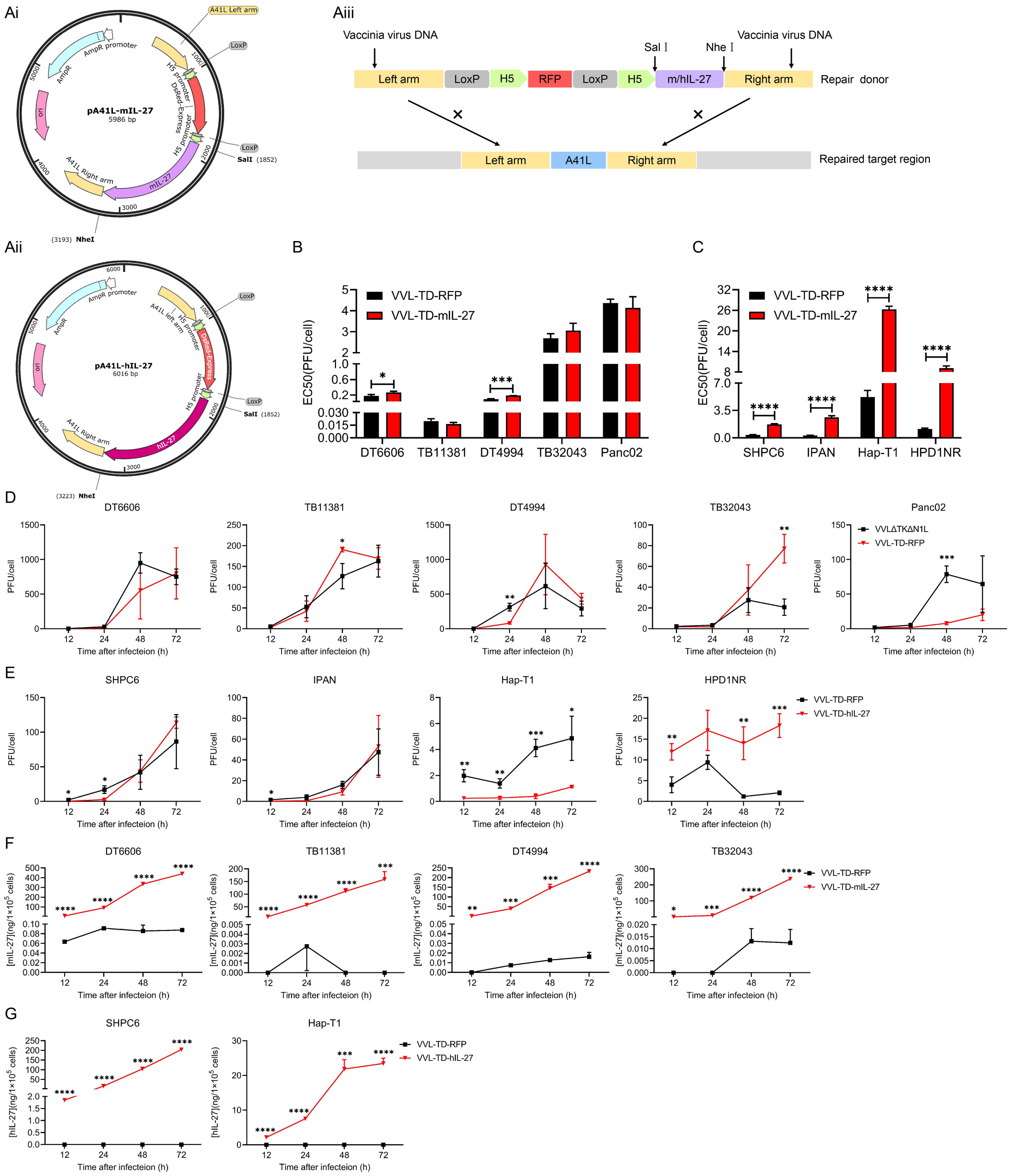

Supplementary figure 4 Construction of VVL-TD-IL-27 and its replication, cytotoxicity and expression of IL-27 *in vitro*. (**A**) Schematic diagram detailing the virus construction. (**Ai, Aii**) Schematic diagram of pA41L-mIL-27 and pA41L-hIL-27 plasmids. (**Aiii**) Schematic of the homologous recombination cassette of the shuttle vector (repair donor vector) and repaired target region in the VV genome of VVLΔTKΔN1L. × denotes homologous recombination. (**B, C**) Cytotoxicity of VVL-TD-RFP and VVL-TD-IL-27 against murine PaCa DT6606, TB11381, DT4994, TB32043, Panc02 cell lines (**B**), and Syrian hamster PaCa SHPC6, IPAN, Hap-T1, HPD1NR cell lines (**C**). Cell death was determined by the MTS assay 144 hours after infection (n=3/group). (**D, E**) Virus replication was determined in murine (**D**) and Syrian hamster (**E**) PaCa cell lines over 72 hours. Virus titers were determined using TCID50 assay (n=3/group). (**F, G**) Mouse and human IL-27

expression was detected in supernatant from the indicated murine (**F**) and hamster (**G**) PaCa cell lines at 12, 24, 48 and 72 hours post infection of 1 PFU/cell VVL-TD-RFP or VVL-TD-IL-27 virus using ELISA (n=3/group). In all cases, the mean ± SD is shown and statistical significance determined using Student's unpaired *t*-test at each time point (n=3). *p<0.05; **p<0.01; ***p<0.001; ****p<0.0001. VVL, *vaccinia virus* Lister strain; PFU, plaque forming unit.

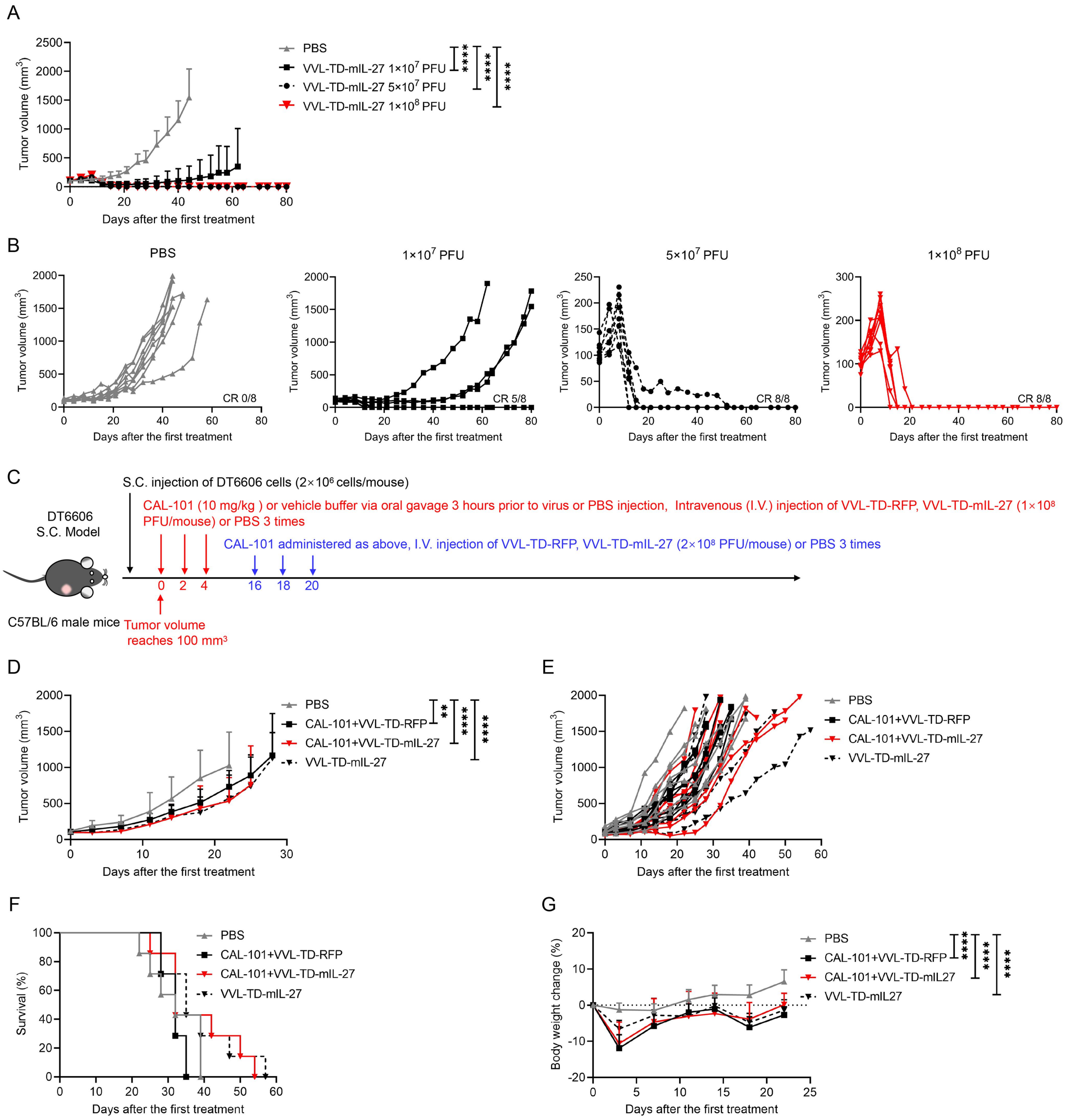

Supplementary figure 5 Optimal dose exploration of VVL-TD-mIL-27 and efficacy assessment after intravenous treatment in the DT6606 subcutaneous tumor model. (**A**) The establishment of the DT6606 tumor model and the treatment schedule were as described in Figure 4A (n=8/group). Tumor growth curves are displayed until the first mouse in each group died. The mean ± SD is shown. A two-way ANOVA with Tukey's multiple comparison post-test was used to compare significance of different groups. Significance at day 44 is shown. ****p<0.0001. (**B**) Individual tumor growth curve of mice. (**C**) Intravenous treatment schedule for the DT6606 subcutaneous

tumor model. DT6606 tumors were established subcutaneously in immunocompetent C57BL/6 mice (n=7/group). When the tumor volume reached 100 mm^3, mice were treated with CAL-101 (10 mg/kg) or vehicle buffer by oral gavage followed 3 hours later with 1×10^8 PFU VVL-TD-RFP, VVL-TD-mIL-27 or PBS via intravenous (I.V.) injection on days 0, 2, 4, and 2×10^8 PFU VVL-TD-RFP, VVL-TD-mIL-27 or PBS on days 16, 18, 20. (**D**) Tumor growth curves are displayed until the first mouse in each group died. (**E**) Individual tumor growth curve of mice. (**F**) Kaplan-Meier survival analysis with log rank (Mantel-Cox) tests was used to assess survival. (**G**) Body weight change curve of mice. The mean ± SD is shown. A two-way ANOVA with Tukey's multiple comparison post-test was used to compare significance of different groups. Significance at day 22 is shown (D, G). **p<0.01; ****p<0.0001. PBS, phosphate-buffered saline; S.C., subcutaneous; I.V., intravenous; VVL, *vaccinia virus* Lister strain; PFU, plaque forming unit.

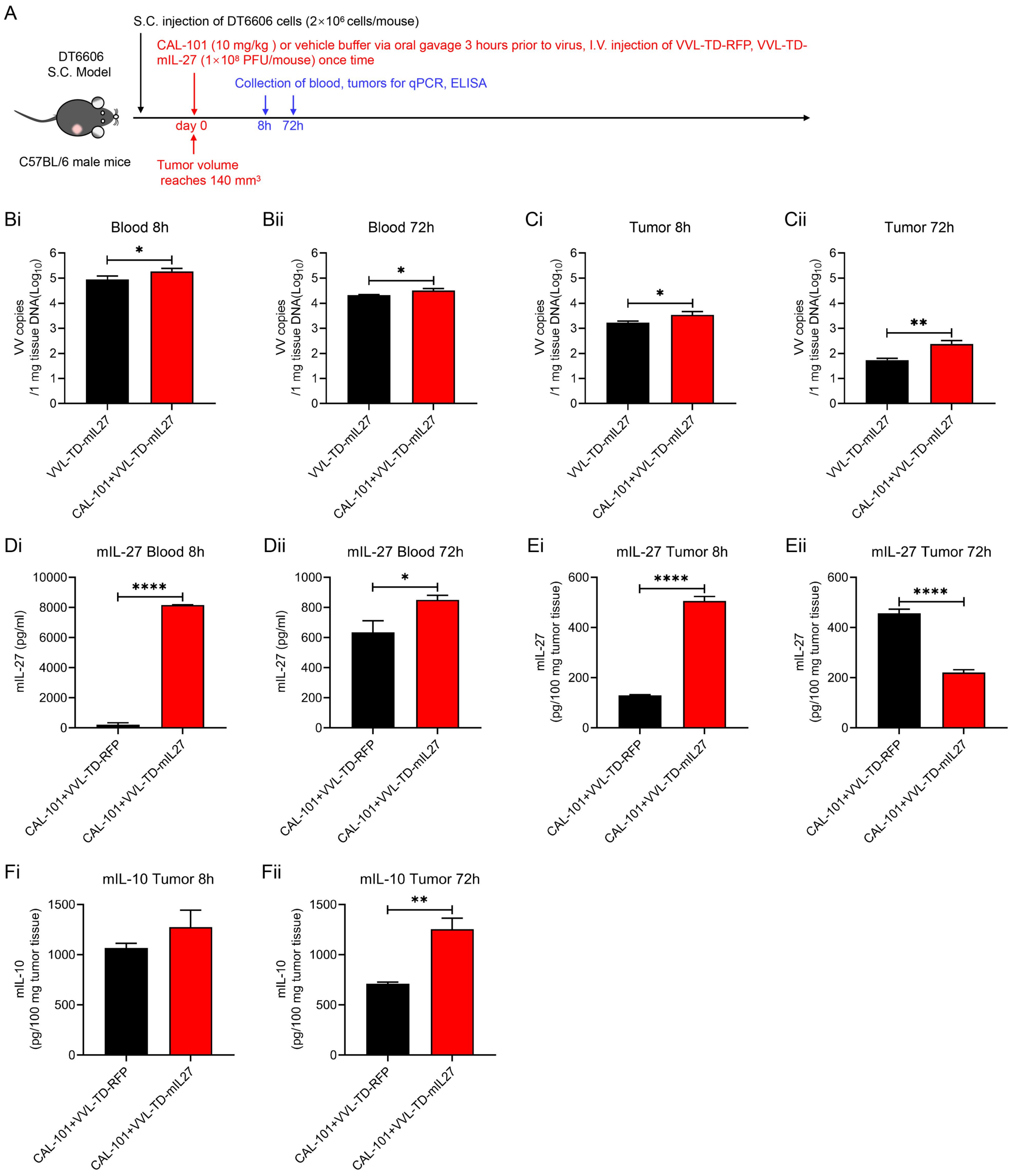

Supplementary figure 6 Detection of viral copy number and cytokine levels in blood and tumor tissues after intravenous treatment. (**A**) Schematic of the experimental protocol. 2×10^6 DT6606 cells were injected subcutaneously into the right flank of 6-7 weeks immune-competent male C57BL/6 mice (n=6/group). When the tumor volume reached 140 mm^3, mice were treated with CAL-101 (10 mg/kg) or vehicle buffer by oral gavage followed 3 hours later with 1×10^8 PFU VVL-TD-RFP or VVL-TD-mIL-27 via I.V. injection once time. At 8 and 72 hours after treatment, blood and tumor tissues were collected, and the virus copy number was detected by quantitative PCR, and the levels of mIL-27 and mIL-10 were detected by ELISA. Viral copy number in blood and tumor tissues (**Bi-cii**), mIL-27 levels in blood and tumor tissues (**Di-Eii**), mIL-10 levels in tumor tissues (**Fi, Fii**) at 8 and 72 hours after I.V. injection. The mean ± SD is shown. Student's unpaired *t*-test was used to analyze difference at each time point (n=3 mice/group/time point). *p<0.05; **p<0.01; ****p<0.0001. VVL, *vaccinia virus* Lister strain; PFU, plaque forming unit; S.C., subcutaneous; I.V., intravenous.

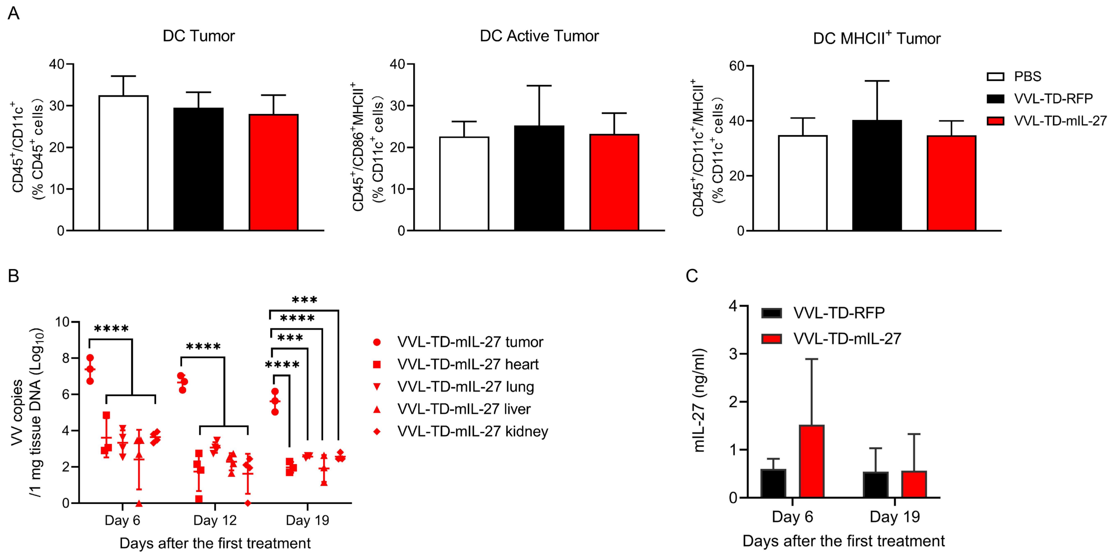

Supplementary figure 7 Analysis of DC subsets in tumors, viral distribution in tumors and organs, and serum IL-27 levels. DT6606 tumors were established and treated as for Figure 5. (**A**) Tumors were collected and analyzed using FC at day 12 after the first treatment. DC were assessed by analyzing $CD11c^+$ population in the $CD45^+$ population, activated DC were assessed by analyzing $CD86^+MHCII^+$ population in the $CD11c^+$ population, $MHCII^+$ DC were assessed by analyzing $MHCII^+$ population in the $CD11c^+$ population. The mean ± SD is shown, and significance was analyzed using a one-way ANOVA with Tukey's multiple comparison post-test (n=3-4 mice/group). (**B**) Tumors, hearts, lungs, livers, kidneys were collected, DNA was extracted, and viral load was analyzed using quantitative PCR shown as virus copy number per mg tissue DNA on days 6, 12, and 19. The mean ± SD is shown. Significance was analyzed using a two-way ANOVA with Tukey's multiple comparisons post-test (n=3-4 mice/group/time point). ***p<0.001; ****p<0.0001. (**C**) Serum was collected and levels of IL-27 were detected by ELISA on days 6 and 19. The mean ± SD is shown. Student's unpaired *t*-test was used to

analyze difference at each time point (n=3-4 mice/group/time point). PBS, phosphate-buffered saline; VVL, *vaccinia virus* Lister strain; DC, Dendritic cell; MHC, major histocompatibility complex.

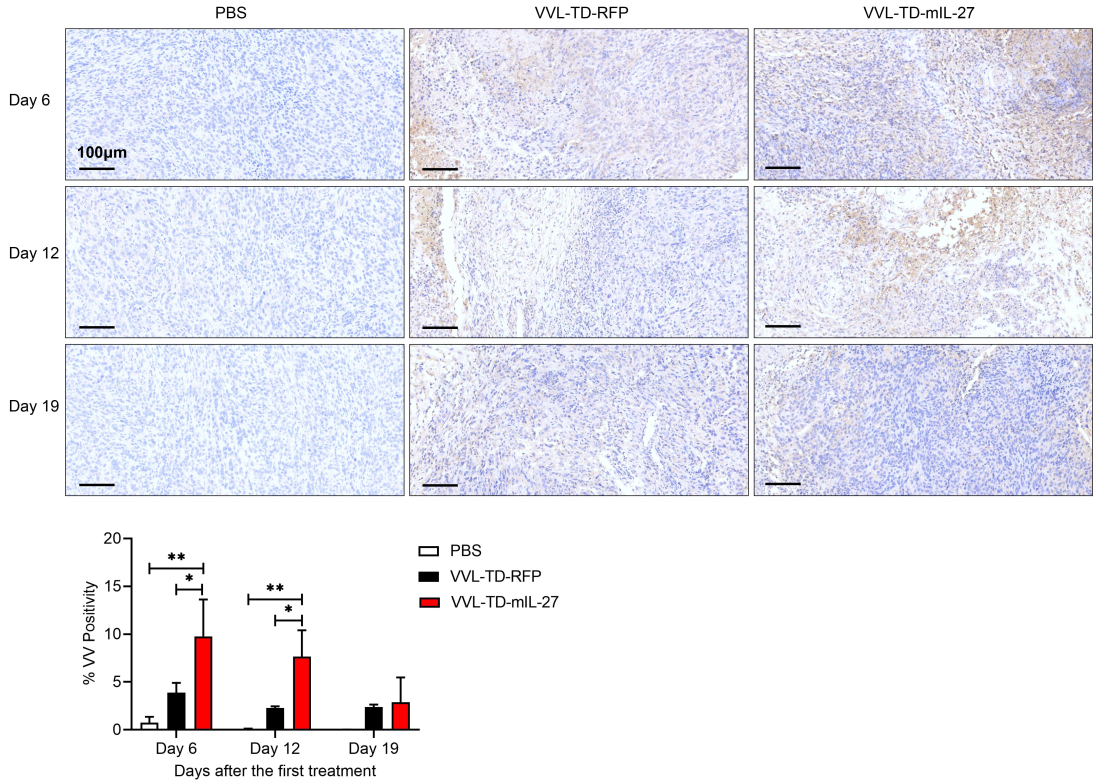

Supplementary figure 8 Analysis of *vaccinia virus* protein expression in tumor tissues by IHC after VVL-TD-mIL-27 treatment. The establishment of the tumor model and the treatment schedule were the same as in Figure 5. (A) Tumors were collected and stained for VV antibodies at days 6, 12, and 19 after the first treatment. Representative IHC images are shown (original magnification ×200). For each tissue sample, greater than 80% of the area was selected and the positive area with VV protein expression was counted using ImageJ. The mean ± SD is shown and significance was analyzed using a one-way ANOVA with Tukey's multiple comparison post-test (n=3 mice/group/time point). *P<0.05; **p<0.01. PBS, phosphate-buffered saline; VVL, *vaccinia virus* Lister strain.

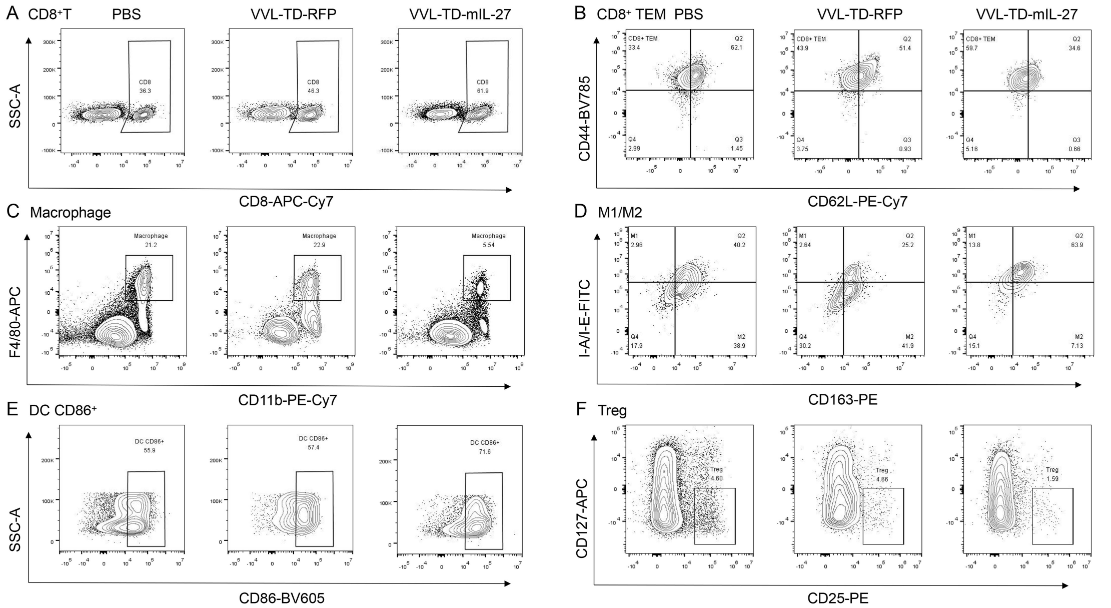

Supplementary figure 9 Representative diagram of FC analysis of immune cells in tumors. DT6606 subcutaneous tumors were harvested and stained with (**A**) anti-CD8; (**B**) anti-CD62L and anti-CD44; (**C**) anti-CD11b and F4/80; (**D**) anti-CD163 and I-A/I-E; (**E**) anti-CD86; (**F**) anti-CD25 and anti-CD127. PBS, phosphate-buffered saline; VVL, *vaccinia virus* Lister strain; TEM, effector memory T cell; M1, M1-polarized macrophage; M2, M2-polarized macrophage; DC, Dendritic cell; Treg, regulatory T cell.

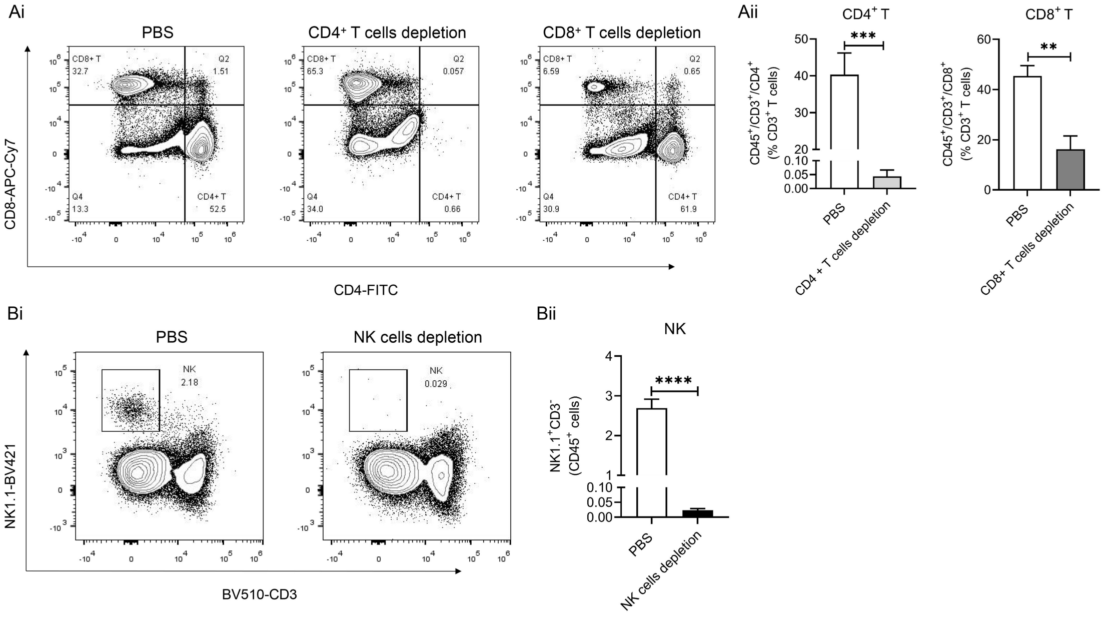

Supplementary figure 10 Antibodies were used to delete CD4⁺ T, CD8⁺ T, and NK cell populations *in vivo*. DT6606 subcutaneous tumors were established in male C57BL/6 mice and viral treatments proceeded as described previously on days 0, 2, and 4. The day before each viral I.T. treatment, anti-mouse CD4⁺ T, CD8⁺ T, and NK antibodies were I.P. injected. (**A**) Depletion of CD4⁺ T and CD8⁺ T cells was confirmed in spleens by FC (**A**i) and the percentage depletion was calculated (**A**ii) at day 2 after the

antibody treatment. (**B**) Depletion of NK cells was also assessed in spleens by FC (**Bi**) and the percentage depletion was calculated (**Bii**). In all cases, the mean ± SD is shown and a student's unpaired *t*-test was used to assess significance (n=3/group). **p<0.01; ***p<0.001; ****p<0.0001. PBS, phosphate-buffered saline; NK, natural killer cell.

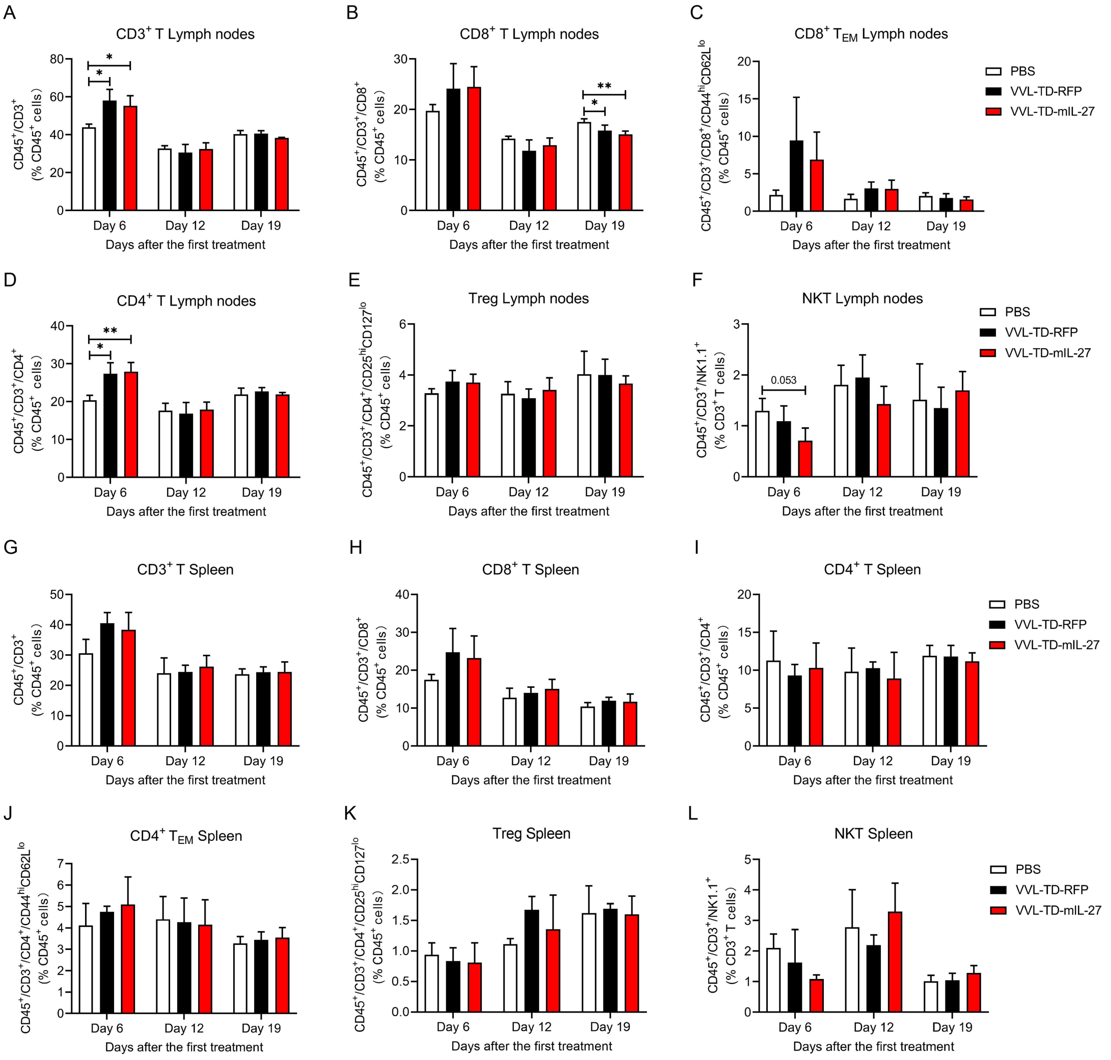

Supplementary figure 11 Analysis of adaptive and innate immune cell populations in lymph nodes and spleens. DT6606 tumors were established and treated as for Figure 5. At days 6, 12, and 19, lymph nodes (**A-F**) and spleens (**G-L**) were harvested and analyzed using FC (n=3-4 mice/group/time point). (**A**) $CD3^+$ T cells were assessed by analyzing $CD3^+$ population in the $CD45^+$ population. (**B**) $CD8^+$ T cells were assessed by analyzing $CD8^+$ populations in the $CD45^+$ population. (**C**) $CD8^+$ TEM was assessed by analyzing $CD44^{hi}CD62L^{lo}$ population in the $CD45^+$ population. (**D**) $CD4^+$ T cells were assessed by analyzing $CD4^+$ populations in the $CD45^+$ population. (**E**) Treg cells were assessed by analyzing $CD25^{hi}CD127^{lo}$ population in the $CD45^+$ population. (**F**) NKT cells were assessed by analyzing $NK1.1^+$ population in the $CD3^+$ population. (**G**) $CD3^+$ T cells were assessed by analyzing $CD3^+$ population in the $CD45^+$ population. (**H**) $CD8^+$ T cells were assessed by analyzing $CD8^+$ populations in the $CD45^+$ population. (**I**) $CD4^+$

T cells were assessed by analyzing $CD4^+$ populations in the $CD45^+$ population. (**J**) $CD4^+$ TEM were assessed by analyzing $CD44^{hi}CD62L^{lo}$ population in the $CD45^+$ population. (**K**) Treg cells were assessed by analyzing $CD25^{hi}CD127^{lo}$ population in the $CD45^+$ population. (**L**) NKT cells were assessed by analyzing $NK1.1^+$ populations in the $CD3^+$ population. In all cases, the mean ± SD is shown, and significance was analyzed using a one-way ANOVA with Tukey's multiple comparison post-test (n=3-4 mice/group/time point). *P<0.05; **p<0.01. PBS, phosphate-buffered saline; VVL, *vaccinia virus* Lister strain; TEM, effector memory T cell; Treg, regulatory T cell; NKT, natural killer T cell.
